# Effect of Different Physical Training Forms on Change of Direction Ability: a Systematic Review and Meta-analysis

**DOI:** 10.1186/s40798-019-0223-y

**Published:** 2019-12-19

**Authors:** Hallvard Nygaard Falch, Håvard Guldteig Rædergård, Roland van den Tillaar

**Affiliations:** Department of Sport Sciences and Physical Education, Nord University, Odins veg 23, 7603 Levanger, Norway

**Keywords:** COD, Effect size, Training form, Specificity

## Abstract

**Background:**

The ability to perform a rapid change of direction (COD) is a critical skill in numerous court- and field-based sports. The aim of this review is to investigate the effect of different physical training forms on COD performance.

**Methods:**

A systematic review of the literature was undertaken using the following databases: PubMed, SPORTDiscus and Google Scholar. Studies were eligible if they met the following criteria: (1) a COD test measuring performance before and after the training intervention, with specific description of the test in terms of length and number of changes in a direction with specified angles, (2) involve training intervention like plyometric, strength, sprint, specific COD training, or a combination of these training forms targeting the lower extremities, (3) the study had to state training background in terms of which sport they participated in and their competitive level and a detailed methodological description. Non-English articles were excluded. Percentage difference and effect sizes were calculated in order to compare the effects of different training interventions.

**Results:**

A range of studies performing plyometrics, strength, sprint, specific COD training, training with post-activation potentiation or a combination of these training forms were examined. The percentage of change and effect size (ES) were calculated. Seventy-four studies met the inclusion criteria, comprising 132 experimental groups and 1652 unique subjects. The review revealed no clear consensus on which training form is optimal to develop COD performance. All training forms resulted in an increase in performance from almost no ES to large ES.

**Conclusions:**

The results of the study indicate that COD ability is a specific skill, whereas the COD task, the sports require determines which training form is the most effective to develop COD ability. Training targeting improvement in COD performance should address the duration of the training in line with which energy system is utilized. The complexity of the COD task with respect to the individual athlete must be considered. Consequently, the number of changes in direction and the angles of the task are relevant when organizing training.

## Key Points

- This meta-analysis highlights the effect of different physical training forms on change of direction ability based in court- and field-based sports.
- In general, strength training is sufficient to develop strength-oriented COD, plyometric training is effective in developing both strength- and velocity-oriented COD, and sprint training is beneficial for velocity-oriented COD.
- Factors such as biological age, experience, type of sport and test assessment also have a large influence on what training form should be performed to enhance COD ability in court- and field-based sports

## Background

In court- and field-based sports, there are different requirements to gain a higher level of performance. An athlete must possess a reasonable level of different skills and capacities. Furthermore, position on the field, playing style and the specific demands of the sport may influence the factors an athlete must cope with [1]. Movement speed is unarguably a central component of many sports. Movement speed can be differentiated into acceleration, maximum speed and agility [2]. The emphasis on movement speed and being able to move quickly in a new direction can provide a physical and tactical advantage over the opponent and is particularly important in sports such as soccer, handball, basketball and rugby [3].

In court- and field-based sports, most of the game is performed at low intensity [4, 5]. Despite the low intensity work, a substantial amount of work is done at high intensity, otherwise known as maximal action, and the ability to repeat maximal actions can be of great relevance [4, 5]. The ability to execute maximal actions in key moments can decide the outcome of a match, such as scoring or preventing a goal [6]. Sprinting, tackling and rapid changes in direction are typical examples of maximal actions and contribute significantly to total energy expenditure [7].

Research has shown that soccer athletes accomplish approximately 700 direction changes during a game of varying intensity, and 600 of these changes in direction are 0–90 ° turns [8]. Roughly 50 of the direction changes in a soccer match are performed at maximal intensity [9].

Data from Póvoas et al. [10] reveal that handball players use a great amount of force during tasks that require changes in direction. Despite the fact that most games are completed at low intensity, the study states that stops, deceleration and changes in direction represent 60% of the physical actions executed in a game. In basketball, 20% of the sprints involve rapid changes in direction [11], which is fewer than the aforementioned sports [8, 9]. Furthermore, data from Duthie et al. [12] suggest that rugby is the sport with the fewest rapid changes in direction compared to the other studies mentioned in this review. Their study revealed that only 16% of all the sprints in rugby included a rapid change in direction. In court- and field-based sports like football, handball, rugby and basketball, it is suggested that the ability to perform rapid changes in direction is an important factor in relation to match outcomes [13]. Due to the specific demands in different sports, pre-planned situations will occur where the ability to perform a rapid change in direction is limited to the athlete’s physical capacity. Therefore, the ability to perform a rapid change in direction is determined by the athlete’s ability to produce a high amount of force in a relatively short time [3].

### Change of Direction

Rapid change of direction (COD) includes an acceleration phase and a deceleration phase, followed by acceleration in a different direction [14]. The acceleration phase is similar to the acceleration in a sprint, which is characterized by lower vertical displacement of centre of mass (COM), which in turn enables greater horizontal ground reaction force to be exerted. The manoeuvre responsible for a COD features eccentric muscle work during braking, followed by concentric muscle work, granting propulsive force [15]. A rapid COD in response to a stimulus has been defined as agility [13, 14, 16, 17]. Without a stimulus, agility is limited only by physical determinants and is therefore termed COD [3]. There are several physical determinants that could influence COD performance, as suggested by Young et al. [17].

### Physical Determinants of COD

**Fig. 1:**
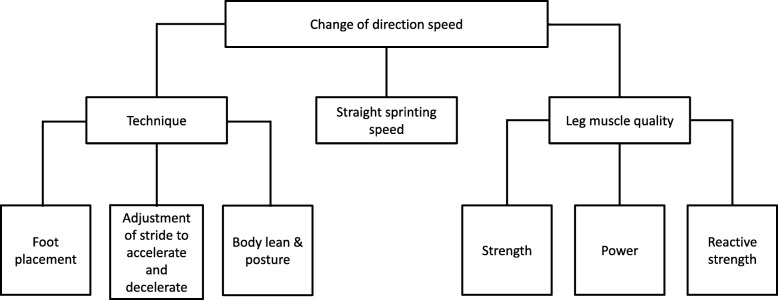
Flow chart illustrating physical determinants when executing rapid CODs. The figure is modified from Young et al. [17], with permission

Different physical determinants affecting COD performance are anthropometrical dispositions and technical qualities, straight sprinting speed, and strength qualities in lower extremities [17] (Fig. 1).

### Anthropometrics

Change of direction performance relies on greater acceleration of body mass and therefore a lower percentage of fat mass, along with great relative strength are desirable [18]. The mechanical forces, expressed via Newton’s laws of motion can explain this. Newton’s first law of motion states that an object (athlete) remains at a constant velocity unless acted upon by a force. This means an athlete must apply force to the ground in order to shift in speed. The necessary force required for a change in speed and direction is dependent on the athlete’s body mass, on velocity when approaching the COD step, and on the angle of direction change [13].

The influence of body mass upon COD is highlighted by Chaouachi et al. [19] who revealed a high correlation between fat-percentage and performance in COD (*r* = 0.8). Athletes with lower percentage of fat mass completed a COD task in less time. A decrease in fat mass or increase in maximal strength without an increase in bodymass increases the athlete’s relative strength, which is beneficial when changing momentum and accelerating in a new direction [13]. Additionally, when changing momentum in a COD task, the athlete is required to rapidly lower the COM for appropriate force production, which is an advantage for shorter athletes, since they commonly possess lower COM than taller athletes. Thereby, they outperform their taller counterparts with significant less completion time in COD tasks [20].

### Technique

Research has shown no clear consensus regarding optimal technique and COD performance. It is assumed that athletes can develop optimal technique through specific COD training themselves since optimal technique is determined by individual anthropometrics and the unique nature of the respective sport [21]. Specific COD training features movement patterns that mimic those performed in competition in terms of COD entry speed, utilization of energy systems, COD angles and number of direction changes [3]. During the COD acceleration phase, a forward shift in COM as a response to forward leaning is required to produce forces horizontal to the ground. A backward lean is necessary to decelerate, and a sideways lean to produce lateral force in order to change direction. Fast postural adjustments and positioning of limbs in COD are essential to produce force in the desired direction and are expected to be very trainable aspects [21]. Furthermore, adjustments of stride to accelerate and decelerate and arm actions are technical factors to be developed when enhancing COD performance [21].

### Reactive Strength, Power and Plyometrics

Due to the restricted time in COD, it is desirable to exert a great amount of force to the ground in a short time. When training with external loads, the rate of force development becomes of relevance. Depending on the COD test, it takes approximately 0.44–0.72 s to develop maximal force [3], which means that athletes should focus on exerting maximal force in this timeframe in exercises aimed at developing COD performance. Reactive strength is one of the subcategories affecting COD performance (see Fig. 1). Reactive strength is the ability to change from eccentric to concentric muscle action as quickly as possible in a stretch-shortening cycle (SSC), as exemplified in a countermovement jump and COD [21]. Plyometric training seeks to exert a high amount of force in a short time. The goal is to increase power output, which is determined by the force and velocity involved in a SSC [22]. Similarities with SSC suggest that plyometric training can facilitate COD performance. Previous studies have revealed moderate to high correlation between different jump exercises and COD tests: *r* = 0.64 [15], *r* = 0.71 [23], *r* = 0.7 [24]. The selection of plyometric exercises can be challenging when training for COD, because the magnitude of distance, speed and direction in COD tasks varies by sport. Previous research has proposed that bilateral and unilateral training in different directions performed at body mass and external load should be targeted when performing plyometric training [13].

### Straight-line Sprinting Speed

**Fig. 2:**
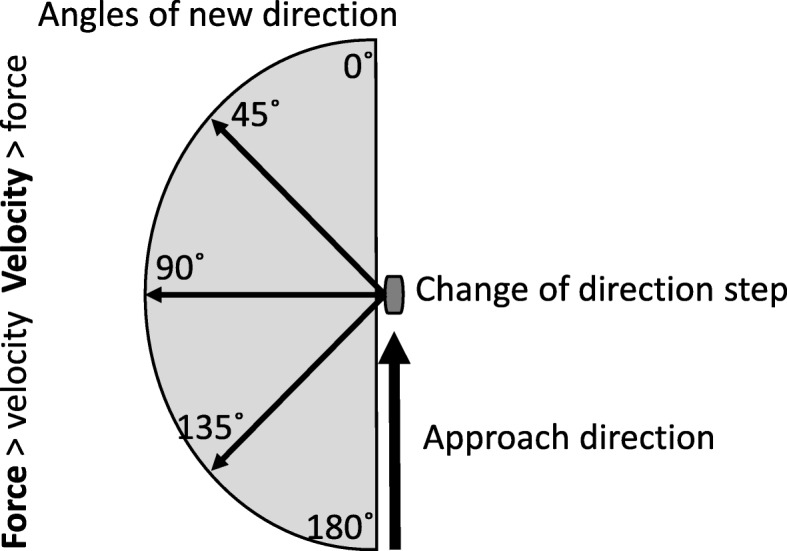
Graphic illustrating mechanical determinants of COD modified from Bourgeois et al. [13], with permission

Previous research has reported moderate to high correlation between straight-line sprinting speed and different COD tests: *r* = 0.3–0.66 [24], *r* = 0.55–0.9 [25], *r* = 0.73 [26], *r* = 0.59 [27]. The correlations will be largely influenced by number of directions, approach speed, angle of direction and total test distance. As suggested by Bourgeois et al. [13], CODs that encompass angles below 90 ° are more velocity-oriented in contrast to angles exceeding 90 °, which are more force-oriented (see Fig. 2). Force-oriented CODs are characterized by a longer completion time at entry of COD, the importance of force capabilities increasing as the magnitude of the COD angle increases. A minor loss of speed and shorter ground contact time characterizes velocity-oriented COD tasks, due to less braking action and smaller direction changes [13]. Most studies that showed a moderate to high correlation between straight-line sprinting speed and COD [25–27] assessed tests with a minimum distance of 15 m and angles of new direction below 90 °, thereby applying velocity-oriented CODs.

A possible explanation for the high correlation values is that the ability to perform fast straight-line sprints is dependent on SSC, while COD and sprints both encompass an acceleration phase. The acceleration phase in COD and straight-line sprint encompass similar technical factors, and an improvement in acceleration ability is likely to improve COD performance over short distances (0–10 m). This is because an athlete should accelerate quickly after the COD step [13]. However, research argues that sprint and COD are independent skills. Two studies finding low correlation between straight-line sprint speed and COD were Jones et al. [16] (*r* = 0.50) and Hewit et al. [28] (*r* = 0.39). Both studies [16, 28] assessed a COD test with a 180 ° angle and a 5–10-m run, which could explain the low correlation between COD and sprint performance, because the COD tests applied in these two studies encompassed strength-oriented CODs [16, 28].

### Strength

In reference to Newton’s laws of motion, one can assume that strength capacity is important due to the requirement to overcome inertia in terms of braking and effectively change momentum in a new direction. Increased maximal strength is important if additional strength gain leads to increased relative strength [13]. It would be reasonable to combine bilateral and unilateral strength exercises since deceleration in COD requires great unilateral eccentric strength along with great unilateral concentric strength before and after the change in direction [13, 18]. Markovic [29] revealed low correlation between COD performance and leg extensor strength. However, the COD tests assessed by Markovic [29] were a 20-yard shuttle run and slalom run with several 45 ° runs, suggesting the tests were velocity-oriented (Fig. 2), which could explain the low correlation (*r* = 0.03–0.44).

Studies have found a moderate to high correlation between strength and COD performance (*r* = 0.4–0.89) [16, 30, 31]. The tests assessed in these three studies were a *T* test or 505-agility test. What these studies had in common were the angles of new direction, which were 90 ° or more, and the short distance for athletes to accelerate. This means that the applied tests were strength-oriented, particularly the 505 test that includes a 180 ° turn (Fig. 2).

Chaouachi et al. [19] found moderate correlation between strength exercises and COD performance (*r* = 0.67–0.69). Their study suggests that eccentric hamstring strength is particularly important when braking during COD tasks. Concentric strength is also important [16], but is a more central aspect when exerting force rapidly during the acceleration phase.

### Complex Training

Complex training involves integration of a strength exercise, typically performed prior to a plyometric exercise. The objective is to increase neuromuscular activity which could increase the performance in a subsequent exercise. It is suggested that greater activation of the fast-twitch muscle fibres occurs, thereby enhancing performance. This is also called the post-activation potentiation (PAP) effect [3]. In Bishop et al. [32], heavy squatting was assessed before performing the proagility COD test (5-10-5 test). They examined optimal resting time for COD performance after heavy squatting. The results showed no tendencies towards positive or negative effects. However, not many studies in this area are performed, which makes it hard to draw any conclusions regarding optimal resting time and exercise protocols that may enhance COD performance.

### Background and Purpose

When considering COD as a multifactorial ability, it is suggested that a combination of training addressing multiple factors can lead to greater performance. Improvement of different physical determinants in COD (Fig. 1) can lead to greater COD performance overall [3]. The ability to perform COD effectively is a key aspect in court- and field-based sports [14]. Therefore, the aim of this review is twofold*:* (1) to investigate how different types of training: strength, plyometric, sprint, specific COD training, training for PAP, or a combination of these approaches can improve COD performance; and (2) to determine the optimal form of training for improvement in COD.

## Material and Methods

### Literature Search

**Fig. 3:**
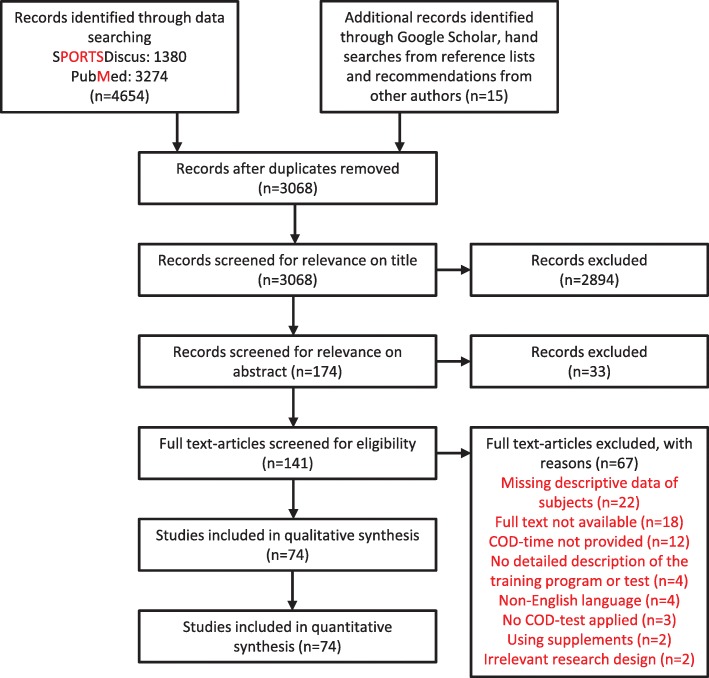
Search results and identification of studies through the different stages of the systematic review

To evaluate the effect of different training interventions upon COD performance, a literature search was completed to structure a meta-analysis. The literature reviewed was attained in August 2019 via the following electronic databases: PubMed, SPORTDiscus and Google Scholar. The following keywords were used in different combinations with change of direction: «Post activation potentiation», «Countermovement jump», «strength», «Plyometric», «Complex», «Training» and «Sprint». Title and abstract were read in order to evaluate the relevance of the articles. During the process of selection of articles, COD tests had to be mentioned in the abstract, with a training intervention targeting performance in lower extremities. The whole paper was read afterwards (Fig. 3)

### Inclusion and Exclusion Criteria

The articles had to contain the following four factors to be included in the study: (1) a COD test measuring performance before and after the training intervention, with specific description of the test in terms of length and number of changes in a direction with specified angles; (2) training intervention performing plyometric training, strength training, sprint training, specific COD training, training for PAP or a combination of these training forms. In addition, workload (volume per training session), number of training sessions per week and number of weeks had to be specified; (3) the study had to state the number of subjects and descriptive statistics concerning individuals’ characteristics (height, mass and age), with their training background in terms of which sport they participated in and their competitive level; and (4) a detailed methodological description conducted with reliable measurement tools. The literature search was not limited by sex and age, and there were no restrictions concerning subjects’ level of performance/physical conditioning. Articles focused on training that did not target lower extremities were excluded.

### Delimitation of Literature

After processing the literature, 74 articles were included. Many training interventions comprised several experimental groups, meaning that different training categories were sometimes contained within the same article. The training interventions included several groups with differences in training programmes, unequal workload, and differences related to performance or sex. These groups were categorised in tables, making it possible to differentiate the subjects’ training improvement based on differences in workload and physical background. A few studies tested the same experimental group in both right and left COD conditions; in these cases, the group will function as two experimental groups.

### Design and Structure of Results Tables

Data from the studies are sorted in Tables 1, 2, 3, and 4, which provide information regarding participants, training interventions, COD tests assessed, improvement in the control group and experimental group from pre- to post-test, and subsequent percentage difference and effect size (ES) in the training intervention. Percentage difference and ES were calculated in order to compare the effects of different training interventions. Effect size was sampled according to Cohen’s d $\left(\ \frac{\mathrm{M}2-\mathrm{M}1}{S}\ \right)$. M2 = mean at post-test, M1 = mean at pre-test. *S* = pooled standard deviation. ES, from 0.01 to 0.2 were defined as very small, wherein values of 0.2–0.5 were considered small ES, values of 0.5–0.8 were considered medium ES, and values of 0.8 or above were considered large ES. Furthermore, ES, 1.2–2 were defined as very large and ES exceeding 2 as huge [106, 107]

**Table 1:** Overview of intervention studies assessing plyometric training and the effect upon COD-performance

Plyometric training									
Reference	Number (*n*) of subjects and mean age	Level and sport	Week tr./tr. per week	Experimental group, improvement (seconds)	Control group, improvement (Seconds)	Improvement, percent (%)	Effect size (ES)	Training intervention, experimental group. (S) series and (R) repetitions per session	COD test 1) Length (meter) 2) Number of CODs 3) Degrees of CODs
Drop jump (DJ)									
Ramírez-Campillo et al. [33]	EG, *n* = 38 Age: 13.2 ± 1.8 CG, *n* = 38 Age: 13.2 ± 1.8	Young men, Soccer	7/2	Pre: 20.3 ± 2.8 Post: 19.6	Pre:20.1 ± 2.7 Post: 20.8	EG: 3.5% CG: -3.5%	EG, ES = 0.26 CG, ES = -0.25	DJ from different heights, S: 2, R: 10	Illinois agility-test 1) 60 m 2) 9 3) 4 × 180 ° and 4 × 60 °.
Ramírez-Campillo et al. [34]	EG, *n* = 25 Age: 13.9 ± 1.9 CG, *n* = 24 Age: 13.7 ± 1.6	High level, young men, soccer	7/2	Pre: 20.4 ± 1.9 Post: 19.8 ± 1.7	Pre: 20.2 ± 1.7 Post: 20 ± 1.6	EG: 2.94% CG: 0.99%	EG, ES = 0,33 CG, ES = 0.12	DJ from fixed height (30 cm). S: 6–9 R: 8–10	Illinois agility-test 1) 60 m 2) 9 3) 4 × 180 ° and 4 × 60 °.
Ramírez-Campillo et al. [34]	EG, *n* = 24 Age: 13.1 ± 1.7 CG, *n* = 24 Age: 13.7 ± 1.6	High level, young men, soccer	7/2	Pre: 20.3 ± 1.7 Post: 19.4 ± 1.3	Pre: 20.2 ± 1.7 Post: 20 ± 1.6	EG: 4.43% CG: 0.99%	EG, ES = 0.6 CG, ES = 0.12	DJ from optimal height. S: 6–9 R:8–10	Illinois agility-test 1) 60 m 2) 9 3) 4 × 180 ° and 4 × 60 °
Thomas et al. [35]	EG, *n* = 6 Age:17 ± 0.4	Men, Jr, soccer	6/2	Pre:2.86 ± 0,5 Post:2.59 ± 0,5	None	EG: 9.44%	EG, ES = 0,54	Vertical DJ (40 cm) 80–120 jumps per session	505-agility test 1) 10 m 2) 1 3) 180 °
Asadi and Ramirez-Campillo [36]	EG, *n* = 6 Age:	College students	6/2	Pre: 11.3 ± 0.7 Post: 10.3 ± 0.8	None	EG: 8.93%	EG, ES = 1.30	Cluster set group. Drop jumps (45 cm). S: 5, R: 20 (2 × 10) with 30–90 sec rest each session.	*T* test 1) 40 m 2) 4 3) 2 × 90 ° and 2 × 180
Asadi and Ramirez-Campillo [36]	EG, *n* = 7 Age:	College students	6/2	Pre: 11.1 ± 1.7 Post: 10.1 ± 0.5	None	EG: 8.32%	EG, ES = 0.84	Traditional group. Drop jumps (45 cm). 2 min rest between sets. S: 5, R: 20.	*T* test 1) 40 m 2) 4 3) 2 × 90° and 2 × 180
Drop jump (DJ) + hurdle jump									
Hammami et al. [37]	EG, *n* = 15 Age: 15.7 ± 0.2 CG, *n* = 13 Age: 15.8 ± 0.2	Men, Jr, Soccer	8/2	Pre: 8.75 ± 0.43 Post: 8.34 ± 0.42	Pre: 8.38 ± 0.33 Post: 8.36 ± 0.45	EG: 4.69% CG:0.24%	EG, ES = 0.964 CG, ES = 0.05	Hurdle jump and DJ, S: 4–10, R: 7–10	S180° 1) 30 m 2) 5 3) 180 °
Drop jump (DJ) + hurdle jump + countermovement jump (CMJ)									
Bouteraa et al. [38]	EG, *n* = 16 Age: 16.4 ± 0.5 CG, *n* = 10 Age: 16.5 ± 0.5	Women, basketball, players	8/2	Pre: 11.3 ± 0.6 Post: 10.6 ± 0.4	Pre: 11.5 ± 0.6 Post: 11.5 ± 0.6	EG: 6.19% CG: 0%	EG, ES: 1.4 CG, ES: 0	Vertical DJ and CMJ, Hurdle jump and zig zag. S: 2–3, R: 10–15. 80–120 jumps per session	Modified Illinois 1) 31 m 2) 7 3) 45–180 °
Drop jump + countermovement jump (DJ + CMJ)									
Hernández et al. [39]	EG, *n* = 7 Age: 10 ± 1.5 CG, *n* = 6 Age: 9.7 ± 2	Young, Men, Basketball	7/2	Pre: 12.3 ± 1.1 Post: 11 ± 1.1	Pre: 12.2 ± 0.9 Post: 11.5 ± 1.1	EG: 10.57% CG: 5.74%	EG, ES = 1.18 CG, ES = 0.7	Non-randomized exercise order. Bilateral and unilateral multidirectional jumps. 47–114 jumps per week.	*T* test 1) 40 m 2) 4 3) 2 × 90 ° and 2 × 180 °
Hernández et al. [39]	EG, *n* = 6 Age: 11 ± 1.7 CG, *n* = 6 Age: 9.7 ± 2	Young, Men, Basketball	7/2	Pre: 12.1 ± 1.1 Post: 10.3 ± 0.7	Pre: 12.2 ± 0.9 Post: 11.5 ± 1.1	EG: 14.88% CG: 5.74%	EG, ES = 2 CG, ES = 0.7	Randomized exercise order. Bilateral and unilateral multidirectional jumps. 47–114 jumps per week.	*T* test 1) 40 m 2) 4 3) 2 × 90 ° and 2 × 180 °
Yanci et al. [40]	EG, *n* = 8 Age: 22.50 ± 5.04	Semi-prof. Men, Soccer	6/1	Pre: 4.92 ± 0.22 Post:4.86 ± 0.25	None	EG: 1.22%	EG, ES = 0,26	Horizontal CMJ and Horizontal DJ, total 180 jump (180 ground contacts per week)	Modified *T* test 1) 20 m 2) 4 3) 2 × 90 ° and 2 × 180 °
Yanci et al. [40]	EG, *n* = 8 Age: 24.63 ± 2.72	Semi-prof. Men, Soccer	6/2	Pre: 4.87 ± 0.25 Post: 4.87 ± 0.20	None	EG: 0%	EG, ES = 0	Horizontal CMJ and Horizontal DJ, total 360 jump (360 ground contacts per week)	1) 20 m 2) 4 3) 2 × 90 ° and 2 × 180 °
Gonzalo-Skok et al. [41]	EG, *n* = 9 Age: 13.2 ± 0.5	Men, Young elite, Basketball	6/2	Pre: 7.21 ± 0.22 Post: 7.01 ± 0.19	None	EG: 2.77%	EG, ES = 0.98	Unilateral horizontal: DJ, long jumps with and without CMJ and repeated jumps. S: 2–5, R: 2–5 60–100 jumps per session	V-Cut test 1) 25 m 2) 4 3) 45 °
Gonzalo-Skok et al. [41]	EG, *n* = 9 Age: 13.3 ± 0.6	Men, Young elite, Basketball	6/2	Pre: 7.37 ± 0.41 Post: 7.21 ± 0.4	None	EG: 2.17%	EG, ES = 0.40	Bilateral vertical: DJ, SJ with and without CMJ, Tuck jump and repeated jumps S: 2–5, R: 2–5 60–100 jumps per session	V-cut test 1) 25 m 2) 4 3) 45 °
Keller et al. [42]	EG, *n* = 12 Age: 14 ± 0.8	Men, Young, Soccer	4/2	Pre: 5.8 ± 0.4 Post: 5.6 ± 0.4	None	EG: 3,45%	EG, ES = 0,50	Vertical: DJ, drop landings, split squat jumps and repeated jumps. S:3–6 R:5–10	Modified *T* test 1) 18 m 2) 4 3) 2 × 90 ° and 2 × 180 °
Ramirez-Campillo et al. [43]	EG, *n* = 8 Age: 12.9 ± 1.9 CG, *n* = 7 Age: 12.6 ± 1.8	Young, Men, Soccer	8/2	Pre: 5.24 ± 0.78 Post: 4.97 ± 0.83	Pre: 5.31 ± 0.58 Post: 5.22 ± 0.55	EG: 5.15% CG: 1.69 %	EG, ES = 0.34 CG, ES = 1,69	Single surface plyometric training. DJ, standing long jump, unilateral CMJ, 180° jump and repeated CMJ. 40–70 jumps per session	1) 10 m 2) 4 3) 60 °
Ramirez-Campillo et al. [43]	EG, *n* = 8 Age: 12.1 ± 2.2 CG, *n* = 7 Age: 12.6 ± 1.8	Young, Men, Soccer	8/2	Pre: 5.41 ± 0.61 Post: 4.93 ± 0.59	Pre: 5.31 ± 0.58 Post: 5.22 ± 0.55	EG: 8.87% CG: 1.69 %	EG, ES = 0.80 CG, ES = 1.69	Combined surface plyometric training. DJ, standing long jump, unilateral CMJ, 180° jump and repeated CMJ. 40–70 jumps per session	1) 10 m 2) 4 3) 60 °
Ramirez-Campillo et al. [44]	EG, *n* = 21 Age: 20.4 ± 2.8 CG, *n* = 21 Age: 20.8 ± 2.7	Men, Soccer, college- level	6/2	Pre: 17.72 ± 0.7 Post: 17.32 ± 0.7	Pre: 17.55 ± 0.6 Post: 17.65 ± 0.7	EG: 2.26% CG: -0.57%	EG, ES = 0.57 CG, ES = -0.15	CMJ bilateral and unilateral, with and without arms + DJ, 80–160 jumps per session	Illinois agility-test 1) 60 m 2) 9 3) 4 × 180 ° and 4 × 60 °
Ramirez-Campillo et al. [44]	EG, *n* = 19 Age: 22.4 ± 2.4 CG, *n* = 19 Age: 20.5 ± 2.5	Women, Soccer, college level	6/2	Pre: 19.48 ± 0.9 Post: 18.73 ± 1	Pre: 19.79 ± 1 Post: 19.93 ± 0,9	EG: 3.85% CG: -0.71%	EG, ES = 0.79 CG, ES = -0.14	CMJ bilateral and unilateral with and without arms + DJ,80–160 jumps per session	Illinois agility-test 1) 60 m 2) 9 3) 4 × 180 ° and 4 × 60 °
Yanci et al. [45]	EG, *n* = 15 CG, *n* = 12Age: 22.5 ± 5.0	Men, amateur, Futsal	6/2	Pre: 2.28 ± 0.09 Post:2.17 ± 0.02	Pre: 2.34 ± 0.15 Post: 2.34 ± 0.08	EG:4.82% CG:0%	EG, ES = 2 CG, ES = 0.00	Vertical DJ, lateral jump and CMJ, 120–176 jumps per week	505-agility test 1) 10 m 2) 1 3) 180 °
Yanci et al. [45]	EG, *n* = 12 CG, *n* = 12 Age: 22.5 ± 5.0	Men, amateur, Futsal	6/1	Pre:2.28 ± 0.09 Post: 2.24 ± 0.06	Pre: 2.34 ± 0.15 Post: 2.34 ± 0.08	EG:1.75% CG:0%	EG, ES = 0.533 CG, ES = 0.00	Vertical DJ, lateral jump and CMJ, 91–176 jumps per week	505-agility test 1) 10 m 2) 1 3) 180 °
Ramirez-Campillo et al. [46]	EG, *n* = 8 Age: 22.8 ± 4.3 CG, *n* = 7 Age: 20.1 ± 1.8	Women, Amateur, Socce	8/1	Pre: 4.94 ± 0.2 Post: 4.57 ± 0.2	Pre: 4.96 ± 0.2 Post: 4.95 ± 0.4	EG: 7.49% CG: 0.20%	EG, ES = 1.85 CG ES = 0.03	DJ, standing long jump, unilateral CMJ, 180° jump and repeated CMJ. 40-70 jumps per week	1) 10 m 2) 4 3) 60 °
Ramirez-Campillo et al. [46]	EG, *n* = 8 Age: 21.4 ± 2.5 CG, *n* = 7 Age: 20.1 ± 1.8	Women, Amateur, Soccer	8/2	Pre: 5.12 ± 0.3 Post: 4.74 ± 0.3	Pre: 4.96 ± 0.2 Post: 4.95 ± 0.4	EG: 7.42% CG: 0.20%	EG, ES = 1.27 CG ES = 0.03	DJ, standing long jump, unilateral CMJ, 180° jump and repeated CMJ. 80–140 jumps per week	1) 10 m 2) 4 3) 60 °
Countermovement jump (CMJ)									
McCormick et al. [47]	EG, *n* = 7 Age:15.71 ± 0.76	Adult women, Basketball	6/2	Right turn: Pre: 23.86 ± 3.13 Post:24.57 ± 2.99 Left turn: Pre:24.00 3.06 ± post:24.14 2.55	None	Right turn: 3.0% Left turn: 0.58%	Right turn, ES = 0.23 Left turn, ES= 0.05	CMJ in sagittal-plane, total 9 exercises. S:4, R:6	Lateral shuffle test to the right and left (6 s, with max number of turns) 1) 2.44 m 2) X 3) 180 °
McCormick et al. [47]	EG, *n* = 7 Age:16.29 ± 0.76	Adult women, Basketball	6/2	Right turn: Pre: 23.00 ± 2.31 Post: 24.57 ± 1.90 Left turn:Pre:22.71 ± 2.22 Post:24.71 ± 2.36	None	Right turn: 6.83% Left turn: 8.81%	Right turn, ES = 0.75 Left turn, ES= 0.87	CMJ in frontal-plane, total 9 exercises. S:4, R:6	Lateral shuffle test to the right and left (6 s, with max number of turns) 1) 2.44 m 2) X 3) 180 °
Ramírez-Campillo et al. [34]	EG, *n* = 10. Age: 11.6 ± 1.4.CG, *n* = 10 Age: 11.4 ± 2.4	Young Men, Soccer	6/2	Pre: 5.31 + 0.27 Post:5.18	Pre: 5.30 + 0.48 Post:5.36	EG:2.5% CG:-1.1%	EG, ES = 0.48 CG, ES = -0.125	Vertical, unilateral and bilateral CMJ. S:3–6, R:5–10	1) 10 m 2) 4 3) 60 °
Ramírez-Campillo et al. [34]	EG, *n* = 10 Age: 11.4 ± 1.9 CG, *n* = 10 Age: 11.4 ± 2.4	Young Men, Soccer	6/2	Pre: 5.36 + 0.45 Post:5.26	Pre: 5.30 + 0.48 Post:5.36	EG:1.9% CG: -1.1%	EG, ES = 0.22 CG, ES = 0.125	Horizontal, unilateral and bilateral CMJ. S3–6: R:5–10	1) 10 m 2) 4 3) 60 °
Ramírez-Campillo et al. [34]	EG, *n* = 10 Age: 11.2 ± 2.3 CG, *n* = 10 Age: 11.4 ± 2.4	Young Men, Soccer	6/2	Pre: 5.36 + 0.52 Post:5.09	Pre: 5.30 + 0.48 Post:5.36	EG:5.1% CG: -1.1%	EG, ES = 0.52 CG, ES = 0.125	Vertical, Horizontal, unilateral and bilateral CMJ. S:2, R:5–10	1) 10 m 2) 4 3) 60 °
Ramírez-Campillo et al. [48]	EG, *n* = 12 Age:11.0 ± 2.0 CG, *n* = 14 Age:11.2 ± 2.4	Young Men, Soccer	6/2	Pre:5.4 ± 0.5 Post:5.19	Pre:5.4 ± 0.6 Post:5.44	EG:3.9% CG: -0.8%	EG, ES = 0.42 CG, ES = -0.07	Bilateral Horizontal and Vertical CMJ. S:2–6, R:5–10	1) 10 m 2) 4 3) 60 °
Ramírez-Campillo et al. [48]	EG, *n* = 16 Age:11.6 ± 1.7 CG, *n* = 14 Age:11.2 ± 2.4	Young Men, Soccer	6/2	Pre:5.3 ± 0.5 Post:4.86	Pre:5.4 ± 0.6 Post:5.44	EG:8.3 CG: -0.8%	EG, ES = 0.88 CG, ES = -0.07	Unilateral Horizontal and Vertical CMJ. S:2–3, R:5–10	1) 10 m 2) 4 3) 60 °
Ramírez-Campillo et al. [48]	EG, *n* = 12 Age:11. ± 6 2.7 CG, *n* = 14 Age:11.2 ± 2.4	Young Men, Soccer	6/2	Pre:5.2 ± 0.6 Post:4.77	Pre:5.4 ± 0.6 Post:5.44	EG:8.3 CG: -0.8%	EG, ES = 0.72 CG, ES = -0.07	Bilateral, unilateral, Vertical and Horizontal jump. S:2, R:5-10	1) 10 m 2) 4 3) 60 °
Ramirez-Campillo et al. [49]	EG, *n* = 12 Age: 22.8 ± 4.3 CG, *n* = 12 Age: 17.1 ± 0.5	High level, Young Men, Soccer	7/2	Pre: 16.58 ± 0.74 Post: 15.87 ± 0.46	Pre: 16.9 ± 0.53 Post: 16.51 ± 0.56	EG: 4.28% CG: 2.31%	EG, ES = 1.18 CG, ES = 0.72	Plyometrics before soccer training. Cyclic and acyclic horizontal and vertical jumps, with left, right and both legs. 104-204 jumps per session.	Illinois agility-test 1) 60 m 2) 9 3) 4 × 180 ° and 4 × 60 °
Ramirez-Campillo et al. [49]	EG, *n* = 14 Age: 21.4 ± 2.5 CG, *n* = 12 Age: 17.1 ± 0.5	High level, Young Men, Soccer	7/2	Pre: 16.55 ± 0.47 Post: 16.43 ± 0.42	Pre: 16.9 ± 0.53 Post: 16.51 ± 0.56	EG: 0.73% CG: 2.31%	EG, ES = 0.27 CG, ES = 0.72	Plyometrics after soccer training. Cyclic and acyclic horizontal and vertical jumps, with left, right and both legs. 104–204 jumps per session.	Illinois agility-test 1) 60 m 2) 9 3) 4 × 180 ° and 4 × 60 °
Thomas et al. [50]	EG, *n* = 6 Age:17 ± 0.4	Men, Jr, Soccer	6/2	Pre:2.89 ± 0.41 Post:2.55 ± 0.41	None	EG:11.76%	EG, ES = 0.83	Vertical CMJ, 80–120 jump per session	505-agility test 1) 10 m 2) 1 3) 180 °
Chtara et al. [51]	EG, *n* = 10 CG, *n* = 10 Age:13.6 ± 0.3	Men, Jr, Soccer	7/1	Pre:7.10 ± 0.23 Post:6.95 ± 0.17	Pre:7.12 ± 0.22 Post:7.07 ± 0.21	EG:2.11% CG:0.70%	EG, ES = 0.75 CG, ES = 0.23	Unilateral, bilateral, lateral, Vertical and Horizontal CMJ, S:2–3, R:8–12	Zig-zag test 1) 20 m 2) 3 3) 100 °
van den Tillaar et al. [52]	EG, *n* = 13 Age: 13.8 ± 0.5	Mixed, Young, handball	6/2	Pre: 6.53 ± 0.75 Post:6.06	None	EG: 7.20%	EG, ES:0.63	Bilateral and unilateral CMJ, max mobilization without bending knees, S:2–3, R:8–25	1) 20 m 2) 5 3) 4 × 135 ° and 1 × 90 °
Bouguezzi et al. [53]	EG, *n* = 15 Age: 11.32 ± 0.27	Young Men, Soccer	8/1	Pre: 12.11 ± 0.83 Post: 11.31 ± 0.76	None	EG: 6.61%	EG, ES = 1.01	Vertical and horizontal CMJ, ankle jumps and zig zag. S: 6–12, R: 8–12. 50–120 jumps per session.	*T* test 1) 40 m 2) 4 3) 2 × 90 ° and 2 × 180 °
Bouguezzi et al. [53]	EG, *n* = 15 Age:12.27 ± 0.33	Young Men, Soccer	8/2	Pre: 12.17 ± 0.98 Post: 11.02 ± 0.45	None	EG: 9.45%	EG, ES = 1.61	Vertical and horizontal CMJ, ankle jumps and zig zag. S: 3–6, R: 7–14. 50–120 jumps per session.	*T* test 1) 40 m 2) 4 3) 2 × 90 ° and 2 × 180 °
Loturco et al. [54]	EG, *n* = 11 Age: 22.2 ± 2.4	Prof. Men, Soccer	5/2	Pre: 6.10 ± 0.20 Post:5.91 ± 0.25	None	EG: 3.11%	E, ES = 0.84	Squat jump with different loads, CMJ vertical and Horizontal jump. S:3–8, R:4–6	Zig-zag test 1) 20 m 2) 3 3) 100 °
Asadi [55]	EG, *n* = 10 Age: 20.2 ± CG, *n* = 10 Age: 20.1 ± 1.5	Division 1. Men, Basketball	6/2	Pre: 12 ± 0.56 Post: 10.97 ± 0.6	Pre:12.15 ± 0.57 Post: 12.57 ± 0.68	EG: 8.58% CG: -3.46%	EG, ES = 1.78 CG, ES = -0.67	Vertical and Horizontal CMJ. S:3, R:15	*T* test 1) 40 m 2) 4 3) 2 × 90 ° and 2 × 180 °
Negra et al. [56]	EG, *n* = 13 ± 0.2 Age: 12.7 ± 0.2 CG, *n* = 11 Age: 12.7 ± 0.2	Young, Men, Soccer	8/2	Pre: 11.1 ± 0.5 Post: 10.3 ± 0.5	Pre: 11.38 ± 0.44 Post: 11.73 ± 0.39	EG: 7.21% CG: -3.08%	EG, ES = 1,60 CG, ES = -0,84	Vertical leap, horizontal jumps, horizontal ankle hop. S:5–8 R:10–15. 50–120 ground contacts per session.	*T* test 1) 40 m 2) 4 3) 2 × 90 ° and 2 × 180 °
Meylan and Malatesta [57]	EG, *n* = 14 Age:13.3 ± 0.6 CG, *n* = 11 Age: 13.1 ± 0.6	Men, Young, Soccer	8/2	Pre: 4.69 ± 0.16 Post: 4.24 ± 0.17	Pre: 4.58 ± 0.22 Post: 4.70 ± 0.25	EG:9.59% CG: -2.62%	EG, ES = 2.73 CG, ES = -0.51	CMJ, repeated jump, ankle jump and Squat-jump, S:2–4, R:6–12	1) 10 m 2) 4 3) 60 °
Chaabene and Negra [58]	EG, *n* = 13 Age: 12.68 ± 0.23	Male soccer players	8/2	Pre: 11.33 ± 0.34 Post: 10.54 ± 0.53		EG: 6.97%	EG, ES = 1.82	Plyometric training, low volume (50–120 contacts per session). CMJs and two-footed ankle-hops.	*T* test 1) 40 m 2) 4 1) 2 × 90 ° and 2 × 180 °
Chaabene and Negra [58]	EG, *n* = 12 Age: 12.72 ± 0.27	Male soccer players	8/2	Pre: 11.58 ± 0.72 Post: 11.23 ± 0.77		EG: 3.02%	EG, ES = 0.47	Plyometric training, high volume (110–220 contacts per session). CMJs and two-footed ankle-hops.	*T* test 1) 40 m 2) 4 1) 2 × 90 ° and 2 × 180 °
Negra et al. [59]	EG, *n* = 17 Age: 12.1 ± 0.5	Male soccer players	8/2	Pre: 11.9 ± 0.4 Post: 11.5 ± 0.1		EG: 3.36%	EG, ES = 1.6	Stable surface. 50–120 contacts per session. Two-footed ankle hop, CMJ. S: 4–6, R: 6–10.	Modified Illinois agility-test 1) 30 m 2) 9 1) 5 × 180 ° and 4 × 45 °
Negra et al. [59]	EG, *n* = 16 Age:12.2 ± 0.6	Male soccer players	8/2	Pre: 11.7 ± 0.3 Post: 11.6 ± 0.1		EG: 0.85%	EG, ES = 0.5	Stable and unstable surface. Two-footed ankle hop, CMJ. S: 4–6, R: 6–10.	Modified Illinois agility-test 1) 30 m 2) 9 3. 5 × 180 ° and 4 × 45 °
Different countermovement jump (CMJ) + hurdle jump									
Faigenbaum et al. [60]	EG, *n* = 14 Age:13.6 ± 0.7	Young Men, 12–15 years	6/2	Pre: 5.6 ± 0.27 Post:5.6 ± 0.25	None	EG: 0%	EG, ES = 0	34 different jumps over 6 weeks. S:1–2, R:6–10 per jump condition	Pro agility shuttle run 1) 18.2 m 2) 3 3) 2 × 90 ° and 1 × 180 °
Fernandez-Fernandez et al. [61]	EG, *n* = 30 CG, *n* = 30 Age: 12.5 ± 0.3	Young Men, Tennis	8/2	Pre: 2.95 ± 0.2 Post: 2.86 ± 0.2	Pre: 2.93 ± 0.1 Post: 2.92 ± 0.1	EG: 3.05% CG: 0.34%	EG, ES = 0.45 CG, ES = 0.1	Different CMJs, Hurdle jump and ankle jump. 6–8 exercises, S:2–4, R:10–15 per week	505-agility test 1) 10 m 2) 1 3) 180 °
Söhnlein et al. [62]	EG: *n* = 18 Age: 13.0 ± 0.8 CG: *n* = 11 Age: 12.0 ± 1.0	Young Men, Soccer	16/2	Pre:11.66 ± 0.79 Post:10.95 ± 0.58	Pre:11.89 ± 0.57 Post:11.71 ± 0.61	EG:6,09% CG: 1,51%	EG, ES = 1.04 CG, ES = 0.31	Unilateral and bilateral jump. Vertical, horizontal, lateral, Hurdle jump, squat jump and ankle jump, 112–350 jumps per week	Hurdle agility run 1) 24 m 2) 7 3.) 90°–180°

Abbreviations: *Tr*:: Training; *CODs*: Change of new directions; *Pre*: Pre-test; *Post*: Post-test; *EG*: Experimental group; *CG*: Control group; *None*: control group not provided

**Table 2:** Overview of intervention studies assessing training for PAP and strength training and the effect upon COD-performance

Training for PAP and strength training									
Reference	Number (*n*) of subjects and mean age	Level and sport	Week tr./tr per week	Experimental group, improvement (Seconds)	Control group, improvement (seconds)	Improvement, percent (%)	Effect size (ES)	Training intervention, experimental group. (S) series and (R) repetitions per session	COD-test 1) Length (meter) 2) Number of CODs 3) Degrees° of CODs
Training for PAP									
Hammami et al. [63]	EG, *n* = 16 Age:16.0 ± 0.5 CG, *n* = 12 Age:16.8 ± 0.2	Men, Jr, Soccer	8/2	Pre: 8.37 ± 0.21 Post: 7.93 ± 0.26	Pre: 8.41 ± 0.33 Post: 8.40 ± 0.35	EG: 5.26% CG: 0.12%	EG, ES = 1.87 CG, ES = 0.03	Half Squat S: 3–5, R: 3–8, 70–90% of 1RM + 4 different squat jumps. Immediate transition for potentiation effect	1) 30 m 2) 5 3) 180 °
Spineti et al. [64]	EG, *n* = 10 Age: 18.4 ± 0.4	Men, Young Soccer	8/3	Pre: 6.93 ± 0.15 Post: 6.97 ± 0.2	None	EG: -0.58%	EG, ES = -0.23	5RM high pull power followed by frontal sprint, knee ups plus frontal sprint and Zig zag sprint. Multiple jumps. R:4–6 + 10 jumps. Immediate transition	RSA 1) 40 2) 1 3) 180°
Hammami et al. [65]	EG, *n* = 14 Age: 16.6 ± 0.3 CG, *n* = 14 Age: 16.6 ± 0.3	Women, Junior, Handball	10/2	Pre: 7.21 ± 0.21 Post: 6.84 ± 0.21	Pre: 7.2 ± 0.34 Post: 7.16 ± 0.35	EG: 5.13% CG: 0.56%	EG, ES = 1.76 CG, ES = 0.12	Heavy strength exercises: Half squats, thigh press, isometric half-squat and calf extension followed by different jumps and 10 m sprints.1–2 min rest for potentiation	Modified *T* test 1) 20 m 2) 4 3) 2 × 90 ° and 2 × 180 °
Alves et al. [66]	EG, *n* = 9 CG, *n* = 6 Age: 17.4 ± 0.6	Young Men, elite- Soccer	6/1	Pre:2.34 ± 0.11 Post:2.31 ± 0.09	Pre:2.37 ± 0.09 Post:2.39 ± 0.15	EG:1.28% CG:-0.84%	EG, ES = 0.3 CG, ES = -0.167	Squat, calf extension, leg extension + high skipping, vertical jump, DJ, S:1, R:1–8	505-agility test 1) 10 m 2) 1 3) 180 °
Alves et al. [66]	EG, *n* = 8 CG, *n* = 6 Age: 17.4 ± 0.6	Young Men, elite- Soccer	6/2	Pre:2.32 ± 0.08 Post:2.32 ± 0.03	Pre:2.37 ± 0.09 Post:2.39 ± 0.15	EG:0% CG:-0.84%	EG, ES = 0 CG, ES = -0.167	Squat, calf extension, leg extension + high skipping, vertical jump, DJ, S:1, R:1–8. Immediate transition for potentiation	505-agility test 1) 10 m 2) 1 3) 180 °
Freitas et al. [67]	EG, *n* = 9 Age: 21.3 ± 4.3	Men, Semi-prof, Basketball	6/2	Pre: 9.45 ± 0.35 Post: 9.16 ± 0.5	None	EG: 3.07%	EG, ES = 0.68	Modified complex training. Half squat and hip trust with optimal loads maximizing power output S: 3–4 R: 3–5. Rest time 2.5 min to enchase potentiation	*T* test 1) 40 m 2) 4 3) 2 × 90 ° and 2 × 180 °
Kontochristopoulos et al. [68]	EG, *n* = 10 CG, *n* = 10 Age: 15.8 ± 1.2	Elite male fencers	8/2	Pre: 5.22 ± 0.31 Post: 5.08 ± 0.27	Pre: 5.38 ± 0.36 Post: 5.42 ± 0.47	EG: 2.68% CG: -0.74%	EG, ES = 0.48CG, ES = -0.1	Half-squat, tuck jumps, step-lunges S: 3, R: 3–10. 1 min rest interval.	4-2-2-4 1) 12 m 2) 3 3) 3 × 180 °
Arazi [69]	EG, *n* = 7 Age: 20.6 ± 0.5	Young Women	6/2	Pre: 13.5 ± 1.0 Post: 13.0 ± 0.9	None	EG: 3.8 %	EG, ES = 0.46	Half Squat, drop jump, knee extension, multiple jumps, knee flexion, zigzag drill, single leg lunge and lunge jump. S: 3, R: 6 (1–2 min rest)	*T* test 1) 40 m 2) 4 3) 2 × 90° and 2 × 180°
Eccentric strength training									
Núñez et al. [70]	EG, *n* = 14 Age: 22.8 ± 2.9	Team, Sport Players	6/2	Dom-leg turn: Pre: 2.7 ± 0.11 Post:2.66 ± 0.12 N-Dom leg turn: Pre:2.68 ± 0.12 Post: 2.64 ± 0.14	None	EG Dom-leg turn: 1.48% EG N-Dom leg turn: 1.49%	EG, ES, Dom-leg turn = 0.35 EG, ES, N-Dom leg turn: 0.31%	Unilateral eccentric overload training. Unilateral lunge on flywheel device. S:4 R.7	505 agility test 1) 10 m 2) 1 3) 180 °
Núñez et al. [70]	EG, *n* = 13 Age: 22.6 ± 2.7	Team, Sport Players	6/2	Dom-leg turn: Pre: 2.68 ± 0.15 Post: 2.66 ± 0.14 N-Dom leg turn: Pre:2.65 ± 0.15 Post: 2.65 ± 0.14	None	EG Dom-leg turn: 0.75% EG N-Dom leg turn: 0%	EG, ES, Dom-leg turn = 0.14 EG, ES, N-Dom leg turn: 0%	Bilateral eccentric overload training. Bilateral half-squat on flywheel device. S:4 R.7	505-agility test 1) 10 m 2) 1 3) 180 °
Siddle et al. [71]	EG, *n* = 8 Age: 20.5 ± 1.55 CG, *n* = 8	Team, Sport Players	6/2	Pre: 4.48 ± 0.12 Post: 4.37 ± 0.11	Pre: 4.52 ± 0.16 Post: 4.62 ± 0.2	EG: 2.46% CG: -2.21%	EG, ES = 0.96 EG, ES = -0.56	Eccentric Nordic hamstring S:2–3 R:5–10	Sprint with COD 1) 20 m 2) 1 3) 180°
Strength training—machine exercises									
Prieske et al. [72]	EG, *n* = 8 Age: 23 ± 3 CG, *n* = 16 Age: 23 ± 2	Men and Women, Young	6/3	Pre: 10.19 ± 0.56 Post: 9.78 ± 0.44	Pre: 10.42 ± 0.91 Post: 10.2 ± 0.86	EG: 4.02% CG: 2.11%	EG, ES = 0.82 CG, ES = 0.25	Power training. Leg press, leg curl, knee extension and calf raise. S:3–5 R:10	*T* test 1) 40 m 2) 4 3) 2 × 90 ° and 2 × 180 °
Strength training—elastic band									
Aloui et al. [73]	EG, *n* = 15 Age: 18.3 ± 0.8 CG, *n* = 15 Age: 18.8 ± 0.8	Men, Junior, Handball	8/2	Pre: 6.18 ± 0.18 Post: 5.7 ± 0.22	Pre: 6.2 ± 0.2 Post: 6.03 ± 0.24	EG: 7.4 % CG: 2.7%	EG, ES = 2.4 CG, ES = 0.8	Different elastic bands with knee and hip extensions. S: 3 R:12–15	Modified *T* test 1) 20 m 2) 4 3) 2 × 90 ° and 2 × 180 °
Strength training—Olympic lifts									
Keller et al. [42]	EG, *n* = 12 Age: 14. ± 0.8	Men, Young, Handball	4/2	Pre: 5.5 ± 0.6 Post: 5 ± 0.3	None	EG: 9.09%	EG, ES = 1.1	Explosive strength. High pull, power clean.	Modified *T* test 1) 18 2) 4 3) 2 × 90° and 2 × 180°
İnce [74]	EG, *n* = 17 Age: 15.63 ± 1.3 CG, *n* = 17 Age: 15.23 ± 1.83	Women, Volleyball Students	6/2	Pre: 11.92 ± 0.87 Post: 11.62 ± 0.61	Pre: 12.7 ± 1.04 Post: 12.71 ± 0.92	EG: 2.52% CG: -0.08%	EG, ES = 0.41 CG, ES = -0.01	Olympic weight lifting. Hang split snatch, hang split clean and split jerk S:3–5 R:5	*T* test 1) 40 m 2) 4 3) 2 × 90 ° and 2 × 180 °
Strength training—squat									
Hammami et al. [63]	EG, *n* = 16 Age: 16.2 ± 0.6 CG, *n* = 12 Age:16.8 ± 0.2	Men, Jr, Soccer	8/2	Pre: 8.38 ± 0.06 Post: 8.09 ± 0.12	Pre: 8.41 ± 0.33 Post: 8.40 ± 0.35	EG: 3.46% CG: 0.12%	EG, ES = 3.22 CG, ES = 0.03	Half Squat S:3–5, R:3–8, 70–90% of 1RM	1) 30 m 2) 5 3) 180 °
Keller et al. [42]	EG, *n* = 9 Age: 14 ± 0.8	Men, Young, Handball	4/2	Pre: 5.5 ± 0.3 Post: 4.8 ± 0.6	None	EG: 12.73%	EG, ES = 1.6	Maximal strength. Squat, deadlift. S:4–6 R: 4–10	Modified *T* test 1) 18 2) 4 3) 2 × 90° and 2 × 180°
van den Tillaar et al. [52]	EG, *n* = 13 Age: 13.8 ± 0.5	Men and women, Young, handball	6/2	Pre:6.78 ± 0.67 Post:6,3	None	EG:7.08%	EG, ES = 0.72	Squat, S: 3,R: 6	1) 20 m 2) 5 3) 4 × 135 ° and 1 × 90 °
Spineti et al. [64]	EG, *n* = 12 Age: 18.4 ± 0.4	Men, Young, Elite Soccer	8/3	Pre:7.11 ± 0.19 Post: 7.04 ± 0.27	None	EG: 0.98%	EG, ES = 0.30	Squat in smith machine, deadlifts, knee extension and flexion, Nordic hamstring, hip adduction and abduction. S:2–4 R:4–15	RSA 1) 40 m 2) 1 3) 180°
Freitas et al. [67]	EG, *n* = 9 Age: 21.3 ± 4.3	Men, Semi-prof, Basketball	6/2	Pre: 9.71 ± 0.67 Post: 9.46 ± 0.3	None	EG: 2.57%	EG, ES = 0.52	Optimal load training. Half squat and hip trust. S: 3–4 R: 3–8	*T* test 1) 40 m 2) 4 3) 2 × 90 ° and 2 × 180 °
Barbalho et al. [75]	EG, *n* = 11 Age: 18.8 ± 0.8 CG, *n* = 12 Age: 19.1 ± 0.9	Men, Young. Soccer	15/3	Pre: 11.5 ± 0.6 Post: 11.7 ± 0.6	Pre: 11.6 ± 0.6 Post: 12.1 ± 0.8	EG: -1.74% CG: -4.31%	EG, ES = -0.33 CG, ES = -0.71	Leg press, squat, leg curl, calf raise. R:4–15 S:2–3	*T* test 1) 40 m 2) 4 3) 2 × 90 ° and 2 × 180 °
Chatzinikolaou et al. [76]	EG: *n* = 12 Age: 14.3 ± 0.7 CG, *n* = 10 Age: 14.1 ± 0.6	Men, Young Soccer	5/4	Left turn: Pre: 8.32 ± 0.33 Post: 8.25 ± 0.33 Right turn: Pre: 8.24 ± 0.37 Post: 8.12 ± 0.37	Left turn: Pre: 8.58 ± 0.33 Post: 8.62 ± 0.36 Right turn: Pre: 8.49 ± 0.35 Post: 8.53 ± 0.34	Left turn: EG: 0.84% CG: -0.47% Right turn: EG: 1.46% CG: -0.47%	Left turn: EG, ES = 0.21 CG, ES = -0.12 Right turn: EG, ES = 0.32 CG, ES = -0.12	Squat, Romanian deadlift, lounges, barbell cleans, kettlebell snatch, box jumps, step ups, 1 leg Romanian deadlift, power bag jerks. S:2–3. R: 6–14.	Arrowhead Test 1) 37.1 m 2) 3 3) 150° × 2 and 110°
de Hoyo et al. [77]	EG, *n* = 11 Age: 18 ± 1	Men, Jr, Soccer, elite- level	8/2	Pre: 4.99 ± 0.10 Post:4.97 ± 0.14	None	EG:0.40%	EG, ES = 0.167	Squat S: 2–3, R: 4–8	Zig-zag test 1) 20 m 2) 3 3) 100 °
Torres-Torrelo et al. [78]	EG, *n* = 12 Age:23.8 ± 2.4 CG, *n* = 10 Age: 24.7 ± 4.7	Men, Futsal	6/2	Pre:8.03 ± 0.29 Post:7.88 ± 0.27	Pre:7.83 ± 0.31 Post:7.96 ± 0.35	EG:1.87% CG: -1.66%	EG, ES = 0.54 CG, ES = -0.39	Squat (45%–48% of 1RM) S: 2–3, R: 4–6	V-Cut test 1) 25 m 2) 4 3) 45 °
Negra et al. [79]	EG, *n* = 13 Age:12.80 ± 0.25 CG: *n* = 11 Age:12.74 ± 0.26	Young Men, Fotall	12/2	Pre: 11.51 ± 0.50 Post:10.92 ± ±0.38	Pre: 11.73 ± 0.59 Post:11.54 ± 0.54	EG: 5.13% CG: 1.62%	EG, ES = 1.34 CG, ES = 0.34	Half-squat S: 4, S: 8–12, core exercises, leg-extensor and flexor exercises.	*T* test 4) 40 m 5) 4 6) 2 × 90 ° and 2 × 180 °
Negra et al. [79]	EG, *n* = 14 Age: 16.1 ± 0.5 CG, *n* = 14 Age: 15.8 ± 0.2	Men, Jr, Soccer	8/2	Pre:6.25 ± 0.26 Post:5.83 ± 0.47	Pre: 6.15 ± 0.25 Post: 6.21 ± 0.24	EG:6.72% CG: -0.98%	E, ES = 1.15 K, ES = -0.24	Squat (70%-90% of 1RM), S:3-5, R:3-8	Modified T-test 1) 20 m 2) 4 3) 2 × 90 ° and 2 × 180 °
Hammami et al. [80]	EG: *n* = 19 Age: 16.2 ± 0.6 CG *n* = 12 Age: 15.2 ± 0.2	Men, Junior Soccer	8/2	Pre: 8.39 ± 0.07 Post: 8.16 ± 0.13	Pre: 8.33 ± 0.29 Post: 8.38 ± 0.35	EG: 2.74% CG: -0.60%	EG, ES = 2.30 CG, ES = -0.60	Half squats S:3-5 R: 3-8 (70-90% of 1RM)	S180° 1) 30 m 2) 4 3) 180°
Panagoulis et al. [81]	EG, *n* = 14 Age: 12.4 ± 0.57 CG, *n* = 14 Age: 12.2 ± 0.5	Men, Young, Soccer	8/3	Right turn: Pre: 17.76 ± 0.4 Post: 17.3 ± 0.5 Left turn: Pre: 18 ± 0.4 Post: 17.4 ± 0.5	Right turn: Pre: 18.4 ± 1.3 Post: 18.4 ± 0.8 Left turn: Pre: 18.3 ± 0.6 Post: 18.7 ± 0.5	Right turn: EG: 2.59% CG: 0.00% Left turn: EG: 3.33% CG: -2.19%	Right turn: EG, ES = 1.02 CG, ES = 0.00 Left turn: EG, ES = 1.33 CG, ES = -0.73	Body mass squat, Nordic hamstring, Body mass RDL, Single leg RDL, squat and side lunges, good mornings, Bulgarian squats. S:1–3 R: 5–8	Arrowhead Test 1) 37.1 m 2) 3 3) 150° × 2 and 110°
Arazi [69]	EG, *n* = 7 Age: 20.7 ± 1.1	Young Women	6/2	Pre: 13.9 ± 0.8 Post: 13.2 ± 0.2	None	EG: 6.4%	EG, ES = 0.97	Half squat, knee extension, knee flexion, single leg lunge. S: 3, R: 6	*T* test 1) 40 m 2) 4 3) 2 × 90° and 2 × 180°
Gonzalo-Skok et al. [82]	EG, *n* = 19 Age: 20.2 ± 1.1	Male team-sports players	8/2	Pre: 9.08 ± 0.37 Post: 8.94 ± 0.31		EG: 1.54%	EG, ES = 0.41	Bilateral vertical strength training. Squats: S: 6, R: 6–10	Average time to complete a 180° COD from 10 m, 20 m and 25 m with both a left and right turn
Speirs et al. [83]	EG, *n* = 9Age: 18.1 ± 0.5	Male rugby players		Pre: 4.61 ± 0.11 Post: 4.53 ± 0.07		EG: 1.74%	EG, ES = 0.89	Unilateral strength training. S: 4, R: 3–6. Split-squat.	Pro-agility shuttle run 1) 18.2 m 2) 3 3) 2 × 90 ° and 1 × 180 °
Speirs et al. [83]	EG, *n* = 9Age: 18.1 ± 0.5	Male rugby players		Pre: 4.71 ± 0.15 Post: 4.64 ± 0.14		EG: 1.49%	EG, ES = 0.48	Bilateral strength training. S: 4, R: 3–6. Bilateral back-squat.	Pro-agility shuttle run 1) 18.2 m 2) 3 3) 2 × 90 ° and 1 × 180 °
Tous-Fajardo et al. [84]	EG, *n* = 12 Age: 17 ± 0.5	Men, Young Soccer	11/1	Pre: 7.09 ± 0.31 Post: 6.7 ± 0.29	None	EG: 5.50%	EG, ES = 1.30	Eccentric overload and vibratory training. Backward lunges, isoinertial hamstring kicks, lateral squats, unilateral squats. 6–10reps	V-Cut 1) 25 m 2) 4 3) 45°

Abbreviations: *Tr*: Training; *CODs*: Change of new directions; *Pre*: Pre-test; *Post*, Post-test; *EG*: Experimental group; *CG*: Control group; *None*: Control group not provided, *Dom-leg*: Dominant leg; *N-Dom leg*: Non-dominant leg; *RM*: Repetition maximum; *RSA*: Repeated sprint ability test; *RDL*: Romanian deadlift

**Table 3:** Overview of intervention studies assessing specific COD-training and sprint and the effect upon COD-performance

COD-training									
Reference	Number (*n*) of subjects and mean age	Level and sport	Week tr./tr per week	Experimental group, Improvement (seconds)	Control group, improvement (seconds)	Improvement, percent (%)	Effect size (ES)	Training intervention, experimental group. (S) series and (R) repetitions per session	COD-test 1) Length (meter) 2) Number of CODs 3) Degrees° of CODs
Specific COD-training									
Teixeira et al. [85]	EG, *n* = 7 Age: 18.71 ± 1.94	Women Futsal	5/2	Pre: 4.52 ± 0.13 Post: 4.57 ± 0.16	None	EG: -1.11%	EG, ES = -0.34	Training with 1 COD (4 × 4)	Shuttle-run 1) 40 m 2) 2 3) 180°
Teixeira et al. [85]	EG, *n* = 7 Age: 18.71 ± 1.94	Women Futsal	5/2	Pre: 4.51 ± 0.2 Post: 4.64 ± 0.16	None	EG: -2.88%	EG, ES = -0.72	Training with 3 CODs (4 × 4)	Shuttle-run 1) 40 m 2) 2 3) 180°
Beato et al. [86]	EG, *n* = 21 Age: 17 ± 0.8	Men, Jr, Soccer	6/2	Pre:4.79 ± 0.13 Post:4.79 ± 0.12	None	EG: 0%	EG, ES = 0	COD- training (45°, 90° and 180° change direction) 84 per week	505-agility test 1) 10 m 2) 1 3) 180°
Young and Rogers [87]	EG, *n* = 12. Age: 17.4 ± 0.7	Australian rules, Football. Men, U-19	7/ 1–2	Pre:8.65 ± 0.45 Post:8.64 ± 0.32	None	EG: 0.12%	EG, ES = 0.026	1–5 COD, under 90°COD, 2–5 m. 4 different exercises per session, 36–48 COD per session	Pre-planned AFL-agility 1) 8 m 2) 1 3) 135 °
Milanovic et al. [88]	EG, *n* = 66 Age = U-19 CG, *n* = 66 Age = U-19	Young Men, Soccer, U-19	4/ 1–2	Pre:7.40 ± 0.33 Post:7.29 ± 0.35	Pre:7.46 ± 0.35 Post:7.49 ± 0.36	EG: 1.49% CG: -0.40%	EG, ES = 0.32 CG, ES = -0.01	Total 48 different exercises for specific COD, a variety of intensities	1) 30 m 2) 4 3) 180 °
Chaouachi et al. [89]	EG, *n* = 12 CG, *n* = 12 Age:14.2 ± 0.9	Young Men, elite- Soccer	6/3	Pre:7.40 ± 0.56 Post:7.03 ± 0.47	Pre:7.74 ± 0.32 Post:7.54 ± 0.30	EG: 5% CG: 2.58%	EG, ES = 0.72 CG, ES = 0.65	S:1–2, R:2–4, 10 m tripping, 505, *T* test and repeated sprint	Zig-zag test 1) 20 m 2) 3 3) 100 °
Chaalali et al. [90]	EG, *n* = 11 Age:14.5 ± 0.9	Young Men, elite- Soccer	6/2	Pre:2.42 ± 0.15 Post:2.34 ± 0.11	Pre:2.52 ± 0.14 Post:2.49 ± 0.09	EG:3.31% CG:1.19%	EG. ES = 0.61 CG, ES = 0.26	2 different CODs each week, with 2–3 completions	505-agility test 1) 10 m 2) 1 3) 180 °
Repeated COD									
Chtara et al. [51]	EG, *n* = 10 CG, *n* = 10 Age:13.6 ± 0.3	Men, Jr, Soccer	7/1	Pre:7.14 ± 0.18 Post:6.86 ± 0.17	Pre:7.12 ± 0.22 Post:7.07 ± 0.21	EG:3.92% CG:0.70%	EG, ES = 1.60 CG, ES = 0.23	505, *T* test, repeated cone-sprint with change direction, S:2, R:2-4	Zig-zag test 1) 20 m 2) 3 100 °
Born et al. [91]	EG, *n* = 19 Age: 14 ± 0.6	Young Men, elite- Soccer	2/3	Pre:17.8 ± 0.3 Post: 17.3 ± 0.5	None	EG: 2.81%	EG, ES = 1.25	Sprint 15 seconds with 180° change direction. S:4,R:5	Illinois agility-test 1) 60 m 2) 9 4 × 180 ° and 4 × 60 °
Born et al. [91]	EG, *n* = 19 Age: 14 ± 0.6	Young Men, elite- Soccer	2/3	Pre:18.2 ± 0.9 Post:17.8 ± 0.6	None	EG: 2.2%	EG, ES = 0.53	Sprint 15 seconds with different change directions S:4, R:5	Illinois agility-test 1) 60 m 2) 9 1) 4 × 180 ° and 4 × 60°
Nakamura et al. [92]	EG, *n* = 10 Age: 14.7 ± 0.5 CG, *n* = 9 Age:14.9 ± 0.4	Young Men, Soccer, High VO2- max	8/2	Pre: 15.3 ± 1.3 Post:14.6 ± 1.5	Pre: 16.5 ± 0.3 Post:16.4 ± 0.4	EG: 4.58% CG: 0.61%	EG, ES = 0.5 CG, ES = 0.29	S:3, R:10, 18 m repeated sprint with two 90° change direction	1) 70 m 2) 3 1) 180 °
Nakamura et al. [92]	EG, *n* = 10 Age: 14.4 ± 0.5 CG, *n* = 9 Age:14.9 ± 0.4	Young Men, Soccer Low VO2-max	8/2	Pre: 16.6 ± 1.4 Post:16.4 ± 1.6	Pre: 16.5 ± 0.3 Post:16.4 ± 0.4	EG: 1.20% CG: 0.61%	EG, ES = 0.13 CG, ES = 0.29	S:3, R:10, 18 m repeated sprint with two 90° change direction	1) 70 m 2) 3 1) 180 °
Taylor et al. [93]	EG, *n* = 7 Age: 24.1 ± 4.1	Semi- prof. Men, Soccer	2/3	Pre: 15.55 ± 0.48 Post: 15.8	None	EG:1.94%	EG, ES = 0.52	Repeated sprint, 10 m + with 180° change direction. S:3–4,R:7	Illinois agility-test 1) 60 m 2) 9 3) 4 × 180 ° and 4 × 60 °
Small-sided games									
Bujalance-Moreno et al. [94]	EG, *n* = 12 Age: 21 ± 5.27 CG, *n* = 11 Age: 20.66 ± 3.39	Men Soccer Semi-Prof/ Amateur	5/2	Pre: 4.83 ± 0.5 Post: 4.5 ± 0.5	Pre: 5.2 ± 0.5 Post: 5.27 ± 0.5	EG: 6.83% CG: -1.35%	EG, ES = 1.32 CG, ES = -0.14	Small-sided games, 18–23 min per session 2vs2 and 4vs4	COD ability test 1) 24 m 2) 4 3) 2 × 45 ° and 2 × 90 °
Coutinho et al. [95]	EG, *n* = 9 Age: 14.2 ± 0.8 CG, *n* = 9 Age: 13.9 ± 0.5	Men Soccer U15	10/2	Pre: 6.74 ± 0.42 Post: 6.34 ± 0.38	Pre: 6.55 ± 0.33 Post: 6.41 ± 0.36	EG: 5.93% CG: 2.14%	EG, ES = 1 CG, ES = 0.41	Small-sided games. 24 min 1vs1, 3vs3, 6vs6	Repeated COD 1) 20 m 2) 4 3) 100°
Coutinho et al. [95]	EG, *n* = 6 Age: 15.8 ± 0.53 CG, *n* = 6 Age: 16.1 ± 0.7	Men Soccer U17	10/2	Pre: 6.45 ± 0.19 Post: 6.12 ± 0.36	Pre: 6.35 ± 0.36 Post: 6.12 ± 0.19	EG: 5.12% CG: 3.62%	EG, ES = 1.20 CG, ES = 0.84	Small-sided games. 24 min 1vs1, 3vs3, 6vs6	Repeated COD 4) 20 m 5) 4 6) 100 °
Paul et al. [96]	Eg, *n* = 12 Age: 16.2 ± 0.8	Men Soccer	4/5	Right turn: Pre: 4.21 ± 0.1 Post: 4.08 ± 0.2 Left turn: Pre: 4.23 ± 0.2 Post: 4.16 ± 0.1	None	Right turn: EG: 3.09% Left turn: EG: 1.65%	Right turn: EG, ES = 0.87 Left turn: EG, ES = 0.47	Small sided games and high intensity training	Modified L-run 1) 20 m 2) 1 3) 90 °
Young and Rogers [87]	EG, *n* = 13 Age: 17.4 ± 0.7	Australian rules, Football. Men, U-19	7/ 1–2	Pre:8.67 ± 0.29 Post:8.74 ± 0.42	None	EG: 0.81%	EG, ES = 0.198	Small-sided game, 4vs4 and 2vs2. 30–45 s	Planned AFL-agility 1) 8 m 2) 1 3) 135 °
Chaouachi et al. [89]	EG, *n* = 12 CG, *n* = 12 Age:14.2 ± 0.9	Young Men, elite- Soccer	6/3	Pre: 7.40 ± 0.56 Post:7.03 ± 0.47	Pre:7.74 ± 0.32 Post:7.54 ± 0.30	EG: 2.47% CG: 2.58%	EG, ES = 0.67 CG, ES = 0.65	Small sided game, 1vs1 (30 sec), 2vs2(1 min) and 3vs3(2 min)	Zig-zag test 1) 20 m 2) 3 3) 100 °
Resisted sprints									
de Hoyo et al. [77]	EG, *n* = 12 Age: 17 ± 1	Men, Jr elite- level Soccer	8/2	Pre: 5.26 ± 0.16 Post:5.28 ± 0.17	None	EG: -0.38%	EG, ES = -0.12	Resisted Sprint with 12,6% of athletes BM, 20 m sprint, S:1, R:4	Zig-zag test 4) 20 m 5) 3 6) 100 °
Prieske et al. [72]	EG, *n* = 10 Age: 23 ± 3 CG, *n* = 16 Age: 23 ± 3	Men and Women, University students	6/3	Pre:11.03 ± 1.23 Post: 10.65 ± 0.58	Pre:10.42 ± 0.91 Post: 10.2 ± 0.86	EG: 3.45% CG: 2.11%	EG: ES = 0.42 CG, ES = 0.25	Resisted sprint exercises with elastic straps on motorized treadmill. S: 3–4	*T* test 1) 40 m 2) 4 3) 2 × 90 ° and 2 × 180 °
Mathisen and Pettersen [97]	EG, *n* = 10 Age:15.5 ± 0.7 CG, *n* = 9 Age: 15.1 ± 0.5	Young Women, Soccer	8/1	Pre:8.23 ± 0.31 Post:7.80 ± 0.33	Pre: 8.04 ± 0.14 Post: 8.01 ± 0.15	EG: 5,22% CG: 0,37%	EG, ES = 1,34 CG, ES = 0,21	Resisted Sprint, over-speed run, sprint with change direction. Total 32 sprints, 15–20 m	Agility course 1) 20 m 2) 4 3) 2 × 90 ° and 2 × 180 °
Straight forward sprint									
Young et al. [98]	EG, *n* = 13 CG, *n* = 10 Age: 24.0 ± 5.7	Volunteer 1-year experience with COD	6/2	Pre: 9.51 ± 0.52 Post: 9.51 ± 0.52	Pre: 9.68 ± 0.67 Post: 9.78 ± 0.66	EG:0% CG:-1.03%	EG, ES = 0 CG, ES = -0.15	Straight forward sprint, with varied distances (20 m – 40 m)	1) 30 m 2) 5 3) 100 °
Repeated sprint									
Chtara et al. [51]	EG, *n* = 12 Age:13.6 ± 0.3 CG, *n* = 10	Men, Jr, Soccer	7/1	Pre:7.15 ± 0.20 Post:6.88 ± 0.14	Pre:7.12 ± 0.22 Post:7.07 ± 0.21	EG:3.78% CG:0.70%	EG, ES = 1.59 CG, ES = 0.23	Repeated sprint with different distances and series, R:2, S:2–4	Zig-zag test 1) 20 m 2) 3 3) 100 °
Taylor et al. [93]	EG, *n* = 8 Age: 24.1 ± 4.1	Semi- prof. Men, Soccer	2/3	Pre: 15.20 ± 0.52 Post: 15.17	None	EG: 0.20%	EG, ES = 0.06	Different numbers of series with repeated sprint, 20 and 30 m S:3–4, R:7	Illinois agility-test 1) 60 m 2) 9 3) 4 × 180 ° and 4 × 60 °

Abbreviations: *Tr*: Training; *CODs*: Change of new directions; *Pre*: Pre-test; *Post*: Post-test; *EG*: Experimental group; *CG*: Control group; *None*: Control group not provided; *BM*: Body mass

**Table 4:** Overview of intervention studies with assessment of combined training and the effect upon COD-performance

Combined training									
Author and year	Number (*n*) of subjects and mean age	Level and sport	Week tr./tr per week	Experimental group, improvement (seconds)	Control group, improvement (seconds)	Improvement, percent (%)	Effect size (ES)	Training intervention, experimental group. (S) series and (R) repetitions per session	COD-test 1) Length (meter) 2) Number of CODs 3) Degrees° of CODs
Plyometric + COD									
Keller et al. [42]	EG, *n* = 12 Age: 14 ± 0.8	Men, Young Soccer	4/2	Pre: 6.5 ± 0.3 Post: 6 ± 0.5	None	EG: 7.69%	EG, ES = 1.25	Horizontal load training. Lateral and forward jumps, Slalom, Zig-zag running and sidesteps drills. S:4–7 R:5	Modified *T* test 1) 18 m 2) 4 3) 2 × 90 ° and 2 × 180 °
de Hoyo et al. [77]	EG, *n* = 9 Age: 18 ± 1	Men, Jr Soccer, elite- level	8/2	Pre: 4.94 ± 0.18 Post:4.94 ± 0.19	None	EG: 0%	EG, ES = 0	Different unilateral CMJ, Hurdle jump and ankle jump + COD, S:1–3, R:3-4	Zig-zag test 1) 20 m 2) 3 3) 100 °
Beato et al. [86]	EG, *n* = 21 Age: 17 ± 0.8	Men, Jr, Soccer	6/2	Pre: 4.72 ± 0.13 Post: 4.73 ± 0.12	None	EG: -0.211%	EG, ES = -0.08	COD (45° and 90°, 180°) + P (DJ + Hurdle jump).36 COD, 60 jumps	505-agility test 1) 10 m 2) 1 3) 180 °
Fernandez-Fernandez et al. [99]	EG, *n* = 8 Age 12.9 ± 0.4	Men, Young Elite, Tennis	5/3	Pre: 2.75 ± 0.16 Post: 2.71 ± 0.16	None	EG: 1.45%	EG, ES = 0.25	Before training. Plyometrics, acceleration, deceleration and ladder drills (COD drills)	505-agility test 1) 10 m 2) 1 3) 180
Fernandez-Fernandez et al. [99]	EG, *n* = 8 Age 12.9 ± 0.4	Men, Young Elite, Tennis	5/3	Pre: 3.03 ± 0.05 Post: 3.05 ± 0.06	None	EG: -0.66%	EG, ES = -0.36	After training. Plyometrics, acceleration, deceleration and ladder drills (COD drills)	505-agility test 1) 10 m 2) 1 3) 180 °
Hammami et al. [100]	EG, *n* = 14 Age: 14.5 ± 0.3 CG, *n* = 14 Age: 14.6 ± 0.2	Men, Young Handball	8/2	Pre: 7.17 ± 0.36 Post: 6.79 ± 0.28	Pre: 7.14 ± 0.21 Post: 7.11 ± 0.23	EG: 5.30% CG: 0.42%	EG, ES = 1.19 CG, ES = 0.14	Lateral jump + COD, lateral hurdle jumps + COD, horizontal jumps + COD, hurdle jumps + COD. 48–144 jumps per week	Modified *T* test 1) 20 m 2) 4 3) 2 × 90 ° and 2 × 180 °
Makhlouf et al. [101]	EG, *n* = 20 Age: 11.29 ± 0.85 CG, *n* = 16 Age: 10.98 ± 0.8	Men, Young Elite Soccer	8/2	Pre: 18 ± 0.6 Post: 17.43 ± 0.6	Pre: 18.38 ± 0.6 Post: 18.28 ± 1	EG: 3.17% CG: 0.54%	EG, ES = 0.95 CG, ES = 0.12	Agility + Plyometric S:1–3 R: 8–15. CMJ, DJ, Horizontal jumps, lateral jumps, ankle jumps, hurdle jumps, rebound jumps. A variety of agility exercises.	Illinois agility-test 1) 60 m 2) 9 3) 4 × 180 ° and 4 × 60 °
Strength + COD									
Torres-Torrelo et al. [78]	EG, *n* = 12 Age: 22.9 ± 5.1 CG, *n* = 10 Age: 24.7 ± 4.7	Men, Futsal	6/2	Pre:7.93 ± 0.36 Post:7.80 ± 0.23	Pre:7.83 ± 0.31 Post:7.96 ± 0.35	EG: 1.64% CG: -1.66%	EG, ES = 0.44 CG, ES = -0.39	Squat (45%–48% of 1RM) S:2–3, R:4–6 + COD: 2–5	V-Cut test 1) 25 m 2) 4 3) 45 °
Strength + COD + plyometric + sprint									
Paul et al. [96]	EG, *n* = 7 Age: 16.4 ± 0.7	Men Young Soccer	4/5	Right turn: Pre: 3.89 ± 0.1 Post: 3.88 ± 0.1 Left turn: Pre: 3.91 ± 0.1 3.88 ± 0.1	None	Right turn: EG: 0.26% Left turn: EG: 0.77%	Right turn: EG, ES = 0.10 Left turn: EG, ES = 0.30	Two strength and power sessions, one speed and agility session and one injury prevention session per week.	Modified L-run 1) 20 m 2) 1 3) 90 °
Strength + COD + sprint									
Gil et al. [102]	EG, *n* = 9 Age: 22.0 ± 2.2	Prof. Men, Soccer	6/1	Pre: 5.83 ± 0.2 Post: 5.44 ± 0,19	None	EG:6.2%	EG, ES = 1.6	Resisted Sprint, S:4, R:6, with elastic bands+ squat jump (60% of 1 RM), S:4–6, R:6	Zig-zag test 1) 20 m 2) 3 3) 100 °
Gil et al. [102]	EG, *n* = 9 Age: 22.8 ± 4.3	Prof. Men, Soccer	6/1	Pre: 5.70 ± 0.1 Post: 5.36 ± 0.14	None	EG:6.1%	EG, ES = 1.6	Sprint without loads S:2–4, R:4, 7 m + squat jump (60% of 1 RM) S:4–6, R:6	1) 20 m 2) 3 3) 100 °
Plyometric + sprint training									
Loturco et al. [54]	EG, *n* = 11 Age: 21.7 ± 2.4	Prof. Men, Soccer	5/2	Pre: 5.93 ± 0.15 Post: 5.78 ± 0.19	None	EG: 2.53%	EG, ES = 0.88	Resisted Sprint, R:6–8(20–30 m) with various loads + squat jump, S:3–8, R:4–6	Zig-zag test 1) 20 m 2) 3 3) 100°
Brocherie et al. [103]	EG, *n* = 8 Age: 17.1 ± 0.3	Youth football players	5/2	Pre: 6 ± 0.1 Post: 5.85 ± 0.14		EG: 2.5%	EG, ES = 1.25	Sprints, CODs, shuttle sprints and high intensive running performed in hypoxia. S: 2–6, R: 3–6.	Repeated-sprint agility 1) 20 m 2) 3 3) 2 × 90 ° and 1 × 180 °
Brocherie et al. [103]	EG, *n* = 8 Age: 17.1 ± 0.2	Youth football players	5/2	Pre: 6.14 ± 0.16 Post: 5.9 ± 0.14		EG: 3.91%	EG, ES = 1.6	Sprints, CODs, shuttle sprints and high intensive running performed in normoxia. S: 2–6, R: 3–6.	Repeated-sprint agility 1) 20 m 2) 3 3) 2 × 90 ° and 1 × 180 °
Plyometric + strength training									
Faigenbaum et al. [60]	EG, *n* = 13 Age: 13.4 ± 0.9	Young Men, 12 – 15 år	6/2	Pre:5.6 ± 0.18 Post:5.4 ± 0.18	None	EG: 3.57%	EG, ES = 1.1	34 different jumps over 6 weeks. S:1–2, R:6–10 per jump- condition + different strength exercises, S:2–3, R:10–12	Pro agility shuttle run 1) 18.2 m 2) 3 3) 2 × 90 ° and 1 × 180 °
Makhlouf et al. [101]	EG, *n* = 21 Age: 11.06 ± 0.75 CG, *n* = 16 Age: 10.98 ± 0.8	Men, Young Elite Soccer	8/2	Pre: 18.16 ± 0.8 Post: 17.69 ± 0.8	Pre: 18.38 ± 0.6 Post: 18.28 ± 1	EG: 2.59& CG: 0.54%	EG, ES = 0.59 CG, ES = 0.12	Strength + Plyometric S:1–3 R:8–15 Variations of squats and lunge exercises, and hip trust.	Illinois agility-test 1) 60 m 2) 9 3) 4 × 180 ° and 4 × 60 °
Ramirez-Campillo et al. [104]	EG, *n* = 9 Age: 17.6 ± 0.5	Men, Young Soccer	8/2	Pre: 5.67 ± 0.29 Post: 5.46 ± 0.2	None	EG: 3.70%	EG, ES = 0.86	Strength and plyometric bilateral group Training knee extensor and flexors. (S:1–3 R:3–10)	Modified *T* test 1) 18 m 2) 4 3) 2 × 90 ° and 2 × 180 °
Ramirez-Campillo et al. [104]	EG, *n* = 9 Age: 17.3 ± 1.1	Men, Young Soccer	8/2	Pre: 5.8 ± 0.29 Post: 5.67 ± 0.16	None	EG: 2.24%	EG, ES = 0.58	Strength and plyometric unilateral group Training knee extensor and flexors. (S:1–3 R:3–10)	Modified *T* test 1) 18 m 2) 4 3) 2 × 90 ° and 2 × 180 °
Plyometric + strength + sprint									
Tous-Fajardo et al. [84]	EG, *n* = 12 Age: 17 ± 0.5	Men Young Soccer	11/1	Pre: 6.94 ± 0.12 Post: 6.9 ± 0.16	None	EG: 0.58%	EG, ES = 0.29	Conventional combined strength training. Strength plyometric and sprint exercises.	V-Cut test 1) 25 m 2) 4 3) 45 °
Otero-Esquina et al. [105]	EG, *n* = 12 CG, *n* = 12 Age: 17.0 ± 1.0	Men, Soccer, Jr- elite	7/1	Pre: 6.66 ± 0.20 Post:6.51 ± 0.18	Pre: 6.63 ± 0.20 Post:6.46 ± 0.20	EG: 2.25% CG:2.56%	EG, ES = 0.79	Squat, S:3, R:4–6, Leg- curl Sledge run, 3–5 R, 20% of BM	V-Cut test 1) 25 m 2) 4 3) 45 °
Otero-Esquina et al. [105]	EG, *n* = 12 CG, *n* = 12 Age: 17.0 ± 1.0	Men, Soccer, Jr- elite	7/2	Pre: 6.80 ± 0.42 Post: 6.46 ± 0.25	Pre: 6.63 ± 0.20 Post:6.46 ± 0.20	EG:5% CG:2,56%	EG, ES = 1.01 CG, ES = 0.85	Squat, S:3, R:4–6 Leg- curl Sledge run, 3–5 R, 20% of BM	1) 25 m 2) 4 3) 45 °
Arazi [69]	EG, *n* = 8 Age: 20.8 ± 0.7	Young Women	6/2	Pre: 14.3 ± 0.9 Post: 13.2 ± 0.9	None	EG: 7.6%	EG, ES = 1.1	Depth jump, multiple jumps, zigzag drill and lunge jump. S: 3, R: 6	*T* test 1) 40 m 2) 4 3) 2 × 90 ° and 2 × 180 °
Arazi [69]	EG, *n* = 7 Age: 20.7 ± 0.7	Young Women	6/2	Pre: 13.4 ± 1.1 Post: 12.3 ± 0.9	None	EG: 8.2%	EG, ES = 1.1	Compound training. Day 1: Depth jump, multiple jumps, zigzag drill and lunge jump. Day 2: Half squat, knee extension, knee flexion, single leg lunge. S: 3, R: 6	*T* test 1) 40 m 2) 4 3) 2 × 90 ° and 2 × 180 °

Abbreviations: *Tr*: Training; *CODs*: Change of new directions; *Pre*: Pre-test; *Post*: Post-test; *EG*: Experimental group; *CG*: Control group; *None*: Control group not provided; *BM*: Body mass

## Results

### Subjects

Among the 74 studies attained, there were 132 experimental groups comprising 1652 subjects. Of these, 1146 subjects were soccer players, 69 handball players, 46 tennis players, 25 Australian rules footballers, 56 futsal players, 110 basketball players, 17 volleyball players and 155 players from unknown sports. There was an average of 12.3 subjects for each experimental group with an average age of 16.5 ± 1.3. Within the experimental groups, 49 groups assessed plyometric training, eight PAP training, 26 strength training, 20 specific COD training, six sprint training, and 23 groups assessed combined training (Fig. 3). Of the 132 experimental groups, 110 assessed males, seven groups were mixed sex and only 17 experimental groups were comprised of females only. Information regarding each consecutive experimental group can be seen in Tables 1, 2, 3, 4.

### Overview

**Fig. 4:**
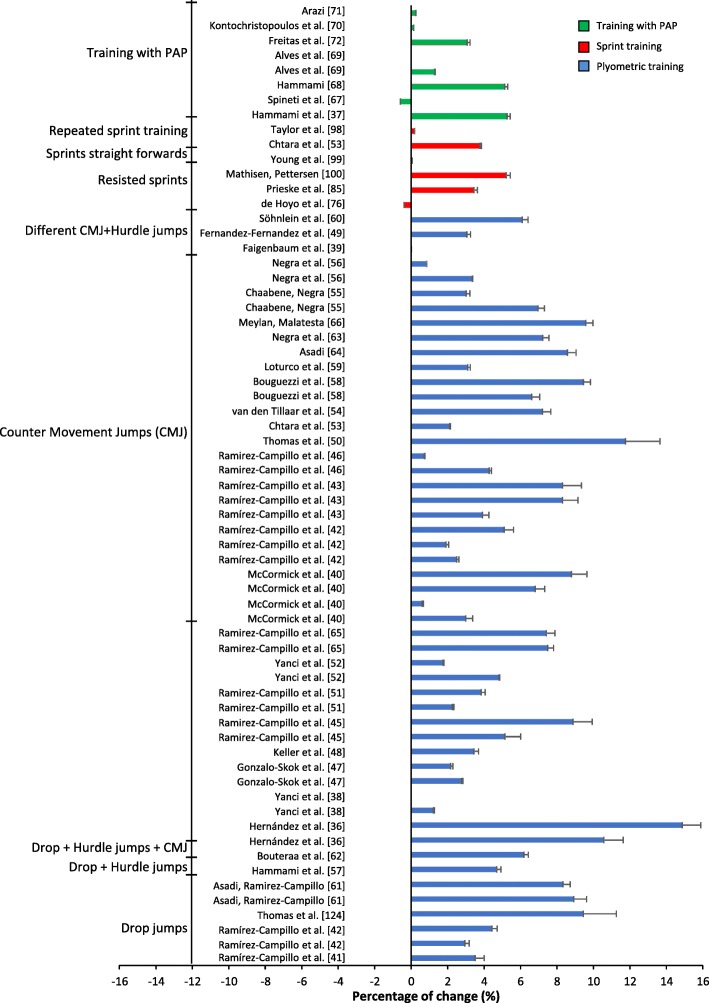
Percentage of change (+SD) after a plyometrics, sprint or PAP training intervention per training group

**Fig. 5:**
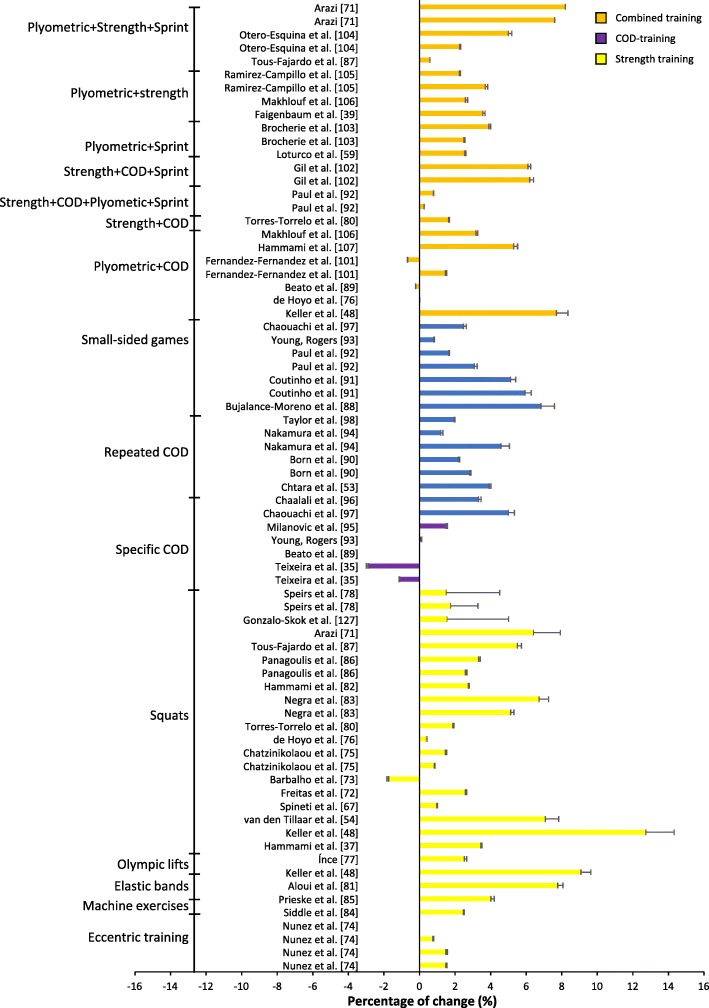
Percentage of change (+SD) after a combined, strength or COD training intervention per training group

**Fig. 6:**
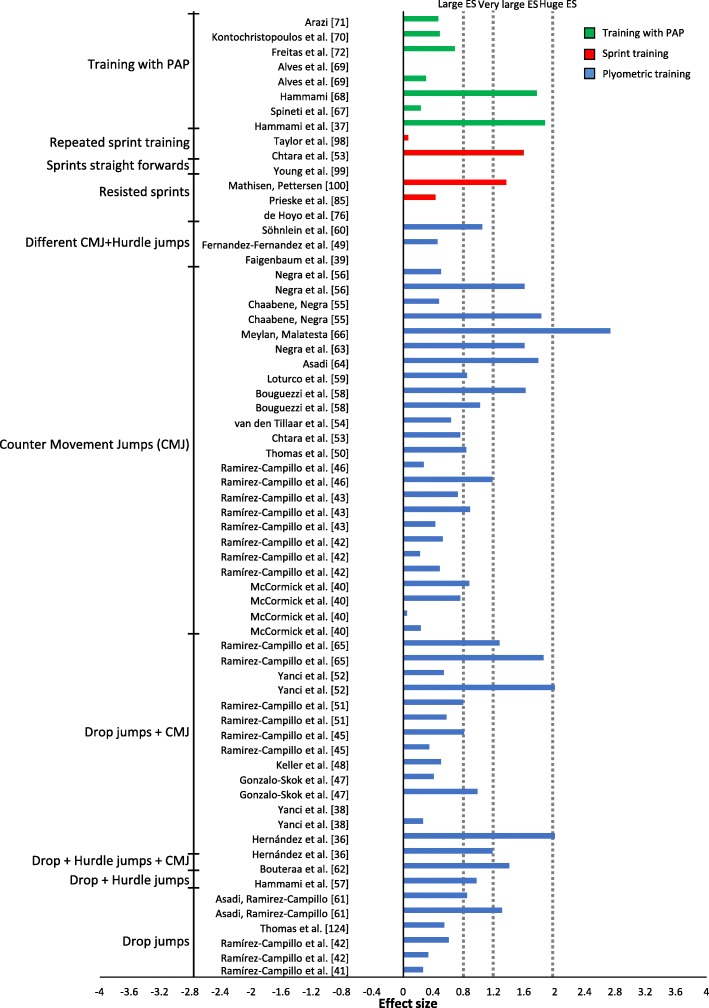
Effect size after a plyometrics, sprint or PAP training intervention per training group

**Fig. 7:**
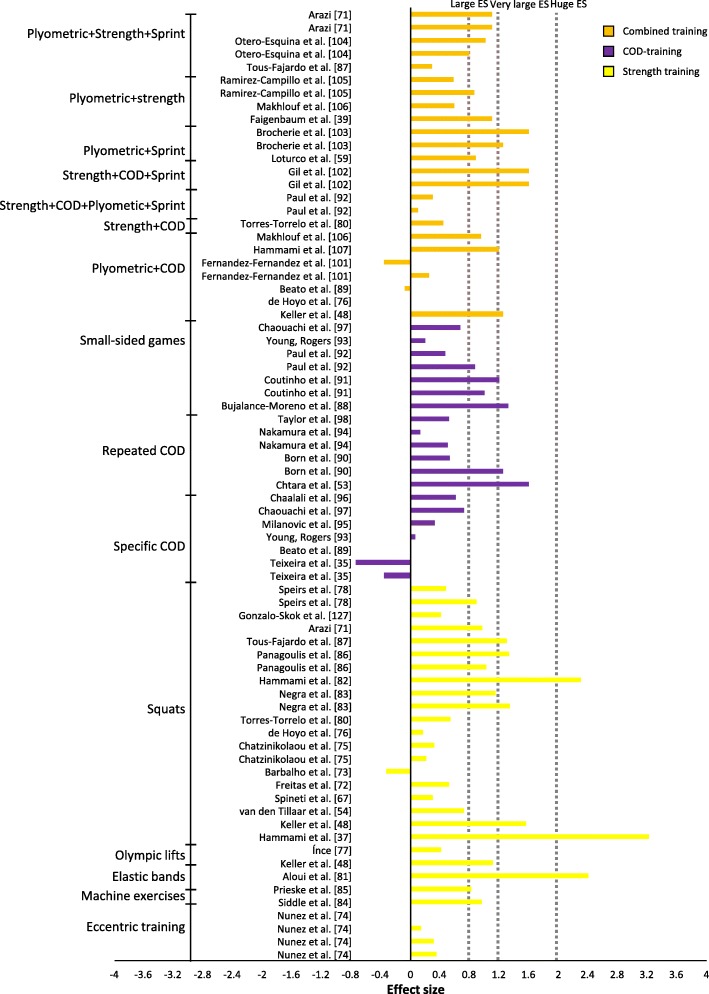
Effect size after a combined, strength or COD training intervention per training group

The percentage change for the studies varied from a ˗2.88% decrease in performance [85] to a 14.88% increase of performance [39] (Figs. 4 and 5); ES varied from no ES to huge ES (Figs. 6 and 7). The intervention that displayed the highest change in percentage assessed plyometric training with drop jumps and countermovement jumps [39]. The study displaying the largest ES assessed strength training with squats [63]. Plyometric training resulted in the highest average percentage change overall for experimental groups (Table 1, Fig. 4).

### Plyometric Training

Studies using plyometric training showed an average percentage change from 0% [40, 60] to 14.88% [39], with no ES to huge ES, respectively (Figs. 4 and 6). The most assessed exercise in the training interventions was countermovement jumps. The study with the highest change in percentage trained in drop jump and countermovement jump [39] (Table 1). Forty-nine experimental groups implementing plyometric training experienced improvement in COD, apart from two groups [40, 60]. Twenty-seven groups revealed very small to medium ES [33, 34, 41–45, 47–52, 58, 59, 61, 108]; eleven groups revealed large ES [36, 37, 39, 41, 47–50, 53, 54, 62]; nine groups revealed very large ES [36, 38, 45, 46, 53, 55, 56, 58, 59]; and two groups showed huge ES [39, 57]. Every experimental group trained twice a week apart from four groups [40, 45, 51, 53]. Experimental groups composed of females all shared effects above the medium ES ( > 0.5), except for one study [47]. Only one study [47] assessed a COD test shorter than 10 m. Thirteen experimental groups assessed COD tests under 90 °, while 16 groups assessed angles above 90 ° and 22 groups performed COD tests above and below 90 °. All subjects in the experimental groups were young, aged ≈24.6 years [40] or younger. Plyometric training led to the greatest improvement in overall percentage difference (experimental 5.26% vs. control 0.98%) in comparison to other training forms.

### Training for PAP and Strength Training

Training for PAP resulted in improvement between ˗0.58% [64] and 5.13% [65] (Fig. 4). Studies assessing PAP trained between one and three times over a period of 6 to 10 weeks. One study exclusively incorporated an exercise that included COD in the training protocol and experienced a decrease in performance [64]. The remaining studies revealed no ES [66], small ES [64, 66, 68, 69] or medium ES [67], followed by two studies revealing very large ES [63, 65] (Fig. 6). The studies revealing very large ES had in common that both experimental groups performed heavy multi-joint exercises followed by different jumps, while one of the experimental groups [65] included an additional 10-m sprint. Training interventions assessing PAP showed a substantial average change in percentage (experimental 2.58% vs. control ˗0.26%).

Studies assessing strength training revealed a change in average percentage between -1.74% [75] and 12.73% [42] (Fig. 5). Most studies trained twice a week for 6 to 8 weeks. The study [42] with the largest improvement in percentage trained with half squats (Table 2). Two experimental groups [70, 75] experienced no improvement. Twelve experimental groups with an average age of 19 years displayed below medium ES [64, 70, 74–77, 82, 83] and trained 2.5 times a week on average. Three experimental groups with an average age of 19.6 years displayed medium ES [52, 67, 78], training twice a week. Twelve experimental groups comprising 16.5-year-old males exceeded large ES [42, 63, 69, 71–73, 79–81, 83, 84] and trained 2.2 times a week on average. All tests applied to measure COD improvement were between 10 and 40 m. Strength training revealed a positive average change in percentage (experimental 3.32% vs. control ˗0.40%).

### Specific COD Training and Sprint Training

Interventions targeting specific training of COD displayed an average change in percentage from ˗2.88% [85] to 6.83% [94], with effect size varying between no ES and very large ES (Figs. 5 and 7). The intervention with the greatest improvement trained in small-sided games [94]. The training interventions lasted from 4 to 10 weeks, with one, two or three training sessions a week (Table 3). All but three experimental groups [85, 86] displayed an improvement in percentage change. Two experimental groups training in repeated COD displayed very large ES [51, 91], followed by four groups assessing small-sided games revealing very large ES [94, 95] and large ES [95, 96]. The remaining groups revealed very small ES [87, 92], small ES [88, 96] and medium ES [89–93]. The effects of specific COD training were relatively low in comparison to control groups (experimental 2.4% vs. control 1.2%).

Experimental groups performing sprints displayed an average percentage change between ˗0.38% and 5.22% (Table 3). Two experimental groups revealed no improvement [77, 98], followed by two groups with very small [93] and small ES [72]. In contrast, two experimental groups revealed very large ES [51, 97]. Sprint training resulted in the following average change in percentage: experimental 2% versus control 0.12%.

### Combined Training

A mixture of different training forms resulted in a percentage change of between ˗0.66% [99] and 8.2% [69] (Table 4, Fig. 5); effects size varied from small ES to very large ES (Fig. 7). The training intervention that produced the greatest results performed plyometrics and sprint training [69]. The training interventions lasted between 5 and 8 weeks, with one to three training sessions a week. All but three experimental groups revealed an improvement post-intervention [77, 86, 99]. Furthermore, five experimental groups displayed very large ES [42, 102, 103], whereas eight groups displayed large ES [60, 69, 100, 101, 104, 105], three groups displayed medium ES [101, 104, 105] and five groups displayed small ES [78, 84, 96, 99] (Fig. 7).

All experimental groups were formed by seven or more subjects. Eleven experimental groups assessed a strength-oriented COD test to measure performance, followed by five velocity-oriented and eight mixed COD tests. Combined training resulted in an increase in COD performance of 3.18% for the experimental group after training interventions versus 0.8% for the control groups.

## Discussion

The main objectives of this review were to examine previous literature on (1) how plyometric, strength, sprint, specific COD training, training for PAP or a combination of these approaches can be performed to improve COD performance, and (2) which form of training is optimal for improvement in COD. Most of the studies revealing large ES employed plyometric training. Studies that included drop jumps and/or countermovement jumps displayed the highest percentage increase in performance (Fig. 4). Strength training resulted in the highest ES [63] (Fig. 7).

### Plyometric Training

Of the experimental groups assessing plyometric training, 23 achieved ES > 0.8 and 28 achieved ES < 0.8 (Fig. 6). Of the 12 experimental groups revealing ES > 0.8, all groups trained twice a week, except Bouguezzi et al. [53]. Previous research on plyometric training frequency indicates that moderate training frequency (approximately two training sessions a week) is optimal in comparison to higher training frequency. de Villarreal et al. [109] suggest that moderate training frequency leads to greater athletic performance in maximal strength and 20 m sprints, which are physical determinants of COD performance.

An important and perhaps surprising finding with useful relevance for strength and conditioning coaches is that younger participants did not reveal greater ES than older participants. The average age of the experimental groups that displayed small ES was 15 years, followed by 15.5 years for those displaying medium ES. Experimental groups revealing ES > 1.2 were 16.1 years old on average. A possible explanation could be that jump exercises assessed in the studies are not properly designed or sufficient in terms of specificity and transferability to the respective COD tests. It is likely that a certain level of motor coordination is required for plyometric training to have a reasonable transfer to COD performance, because plyometric training can be technically challenging for younger athletes [13]. Older athletes commonly possess a greater level of strength [110]. It must also be considered that younger athletes gain greater strength after puberty because of more muscle mass [111], increased level of testosterone [112] and increased motor control [113, 114]. Previous research points out that physically stronger athletes will benefit more from plyometric training compared to weaker athletes [13]. It is also known that females have less muscle mass and more fat mass and thereby less relative strength compared to males [110], a possible handicap when utilizing plyometric training.

One of the 16 experimental groups revealing ES below medium was composed of females with an average age of 15.7 years. Since six groups of females trained in plyometrics, it would be expected that more groups of females would achieve low ES. The results are more surprising since four experimental groups revealed large ES, as opposed to 19 for males. The experimental groups of females who displayed large ES were 19.2 years old on average versus 15.4 years for males. Therefore, the surprising COD results between sexes could be linked to the differences in biological age, although this was not investigated. Alternatively, increased muscle-tendon stiffness following plyometric training could explain some of the findings. Tendon stiffness is an important factor for performance improvement in actions that include SSC [115]. To our knowledge, it is unclear how adaptations in muscle-tendon vary by sex following plyometric training. Theoretically, the effects of females’ increased COD performance could be due to similar tendon stiffness adaptations between sexes, in contrast to males’ advantage in gaining muscle mass.

In all COD tests where the experimental groups revealed ES > 0.8, the test was between 10 and 40 m, except for one group [49]. The duration and intensity of the test will determine the release of metabolic energy during COD. While in the end, high-energy phosphates are the main source of energy during CODs, with increasing duration (several CODs over a longer period of time) the predominant pathway for energy will change [116]. Within the groups that displayed small and medium ES, three and four groups respectively used COD tests of over 40 m [34, 41, 43–45, 49, 61, 96]. These groups performed short duration plyometric exercises, but the applied COD tests lasted over 15 s. The relatively low ES achieved in these groups can partly be explained by different demands in terms of energy release in plyometric training in comparison to the applied COD tests: longer COD tests will challenge the different energy systems to a greater extent [117]. Other factors could be technique while executing the COD test, familiarity with the COD test, neuromuscular and psychomotor statuses on test day, etc.

There were no consistent trends regarding studies addressing strength-oriented, velocity-oriented CODs or a mixture of both in terms of effect sizes. However, three experimental groups [46, 57] implementing velocity-oriented CODs achieved ES > 1.2 versus one group [45] that used a force-oriented COD test. The remaining groups revealing ES > 1.2 used a mixture of different COD angles. An important aspect of plyometric training is to enhance relative strength and rapid exertion of force to the ground [30]. These results suggest that plyometric training may be more effective for athletes that need to develop both velocity-oriented and strength-oriented COD performance.

All plyometric training interventions included countermovement jumps or drop jumps. The reason why some groups failed to achieve large ES can be more complex than in the previously stated arguments. Drop jumps could have been performed with suboptimal drop height or poor technique [13]. Data from Ramirez-Campillo et al. [43] revealed that soccer athletes performed better from optimal drop jump height (medium ES) versus fixed drop jump height (small ES). Furthermore, countermovement jumps could have been performed with a lack of specificity in terms of depth and not enough emphasis on fast muscle actions in eccentric and concentric work [117]. The benefit of drop jumps in comparison to countermovement jumps is that the eccentric phase requires a greater amount of force to be exerted in the lower extremities. Furthermore, eccentric hamstring strength, among other factors, has proved to be important during deceleration in COD [20]. Data from this review show that drop jumps along with countermovement jumps seems to be an optimal approach compared to training that involves a single exercise approach. Brughelli et al. [117] suggest that plyometric training aimed at developing COD ability should be performed both bilaterally and unilaterally, whereas force exerted to the ground is completed in vertical, horizontal and lateral directions.

### Training for PAP

Seven studies formed by eight experimental groups used PAP to develop COD performance. Only two studies [64, 69] implemented an exercise that included a COD in the training protocol. Spineti et al. [64] involved zig-zag sprints after 5-RM high pulls, the experimental group experienced a decrease in COD performance. Olympic lifts are unarguably a technically challenging exercise: if the technique is not highly developed, it could hypothetically restrict the activation of high-threshold muscle fibres and counteract the PAP effect upon the following exercises. Arazi [69] also included zig-zag sprint as part of the program and displayed small ES in untrained individuals. The lack of improvements for studies including PAP could also be explained by relatively high training frequency. Alves et al. [66] had two experimental groups performing the same training protocol, but they differed in training frequency. The group training twice a week experienced no ES and the group training once a week experienced small ES in COD improvement. While the differences between the groups were minimal, the results suggest that higher training frequency (≥ 2) may not be suitable when training for PAP. The experimental groups included several sets of plyometric training, and previous research suggests that moderate training frequency is optimal [109]. It is also very well known that strength training with repetitions close to 1RM causes substantial neuromuscular fatigue. The results are therefore unsurprising.

The lack of improvement could also be explained by the fact that there is no clear consensus regarding rest time in relation to PAP effect, in addition to the individual muscle fibre compositions, which can determine the PAP effect [118].

The remaining groups that achieved marked improvements in COD performance used half squats before plyometric exercises. The half squat is performed with a shorter range of motion, compared to the exercises used in those studies with poor results. Previous research [119] reveals that squats performed with a shortened range of motion improve performance in jumps and sprints in comparison to deeper squats. Two of the groups revealing great improvement used the *T* test with 90 ° and 180 ° direction changes. Considering that force- and velocity-oriented CODs are determined by both COD angle and approaching velocity [13], the 180 ° direction change in the *T* test is arguably velocity-oriented, due to sideways running prior to the COD which causes a relatively low velocity when entering the 180 ° angle of direction change. Less vertical displacement of COM is expected in velocity-oriented CODs, which means a shorter range of motion, as in half squats. Theoretically, and based on these findings, the effect of studies implementing half squats could be threefold:

1. Relatively high number of reps in squat sets could have an isolated effect on the subject’s strength gain, thus making plyometric training more effective in the subsequent training session.
2. Considering that an increase in neural drive occurs at the specific angles trained [120], it is reasonable that the acute PAP effect is joint angle-specific, giving half squats an advantage over deep squats at generating increased neural drive for the exercises performed with similar joint angles within the same training session.
3. If half squats, in fact, encompass similar joint angles as in the COD test, new strength gain from the exercise is likely to transfer to the applied COD test post-intervention due to the principle of specificity as introduced by Henry [121].

A trend in PAP training is the lack of specific exercises: only one study [64] included force exertion in a lateral direction, braking or reacceleration in the training programme. No clear conclusion can be drawn on the effect of this form of training upon COD performance because of the low number of studies. Future research should expand the knowledge regarding PAP training in relation to COD performance in general, and how exercises performed at specific joint angles influence the PAP effect.

### Strength Training

Of 29 experimental groups, 14 displayed ES > 0.8 (average age 16.5 years), whereas three of these studies revealed huge ES (> 2); all trained twice a week for 8 weeks. The tests applied to measure COD performance included at least one strength-oriented COD. Two studies revealing huge ES trained using half squats [63, 80] at 70–90% of 1RM, and one study trained with elastic bands [73]. Aloui et al. [73] implemented elastic band training, which consisted of knee and hip extension exercises, where the participants progressively increased the number of sets and elastic band resistance during the training period. The group training with elastic bands were on average 18.3 years old, while both the groups training with half squats were on average 16.2 years old. The larger improvements can be explained by the role of puberty in physical development and the fact that most gains due to neurological adaptations following a maximal strength programme are achieved in the first weeks [122–124].

The remaining experimental groups displaying ES > 0.8 trained with Olympic lifts [42] and different squat variations, one study training in the Nordic hamstring exercise [71]. The study implementing the eccentric Nordic hamstring exercise increased COD performance by 2.46%, supporting statements in previous research regarding the importance of eccentric hamstring strength during the deceleration phase in COD [20].

The experimental groups showing medium ES trained with squats twice per week. In contrast, the groups that had below medium ES trained with squats and other multi-joint exercises, Olympic lifts and eccentric strength exercises 2.5 times a week on average. These groups did not differ in age and the COD tests to measure performance were mostly strength-oriented for all groups. The only difference between the medium ES and < 0.5 ES group was some variation in exercise selection and training frequency.

Monotonous training with squats can be problematic: while it demands great force exertion, no propulsion occurs as in COD. During a traditional squat, force is produced vertically, but deceleration and acceleration in COD require vertical-horizontal exertion of force [21].

These results suggest that a moderate strength training frequency seems to be optimal and squats need to be combined with motions that create horizontal exertion of force to give reasonable transfer to COD performance. Furthermore, the group displaying medium ES was three years older than the groups displaying ES > 0.8. Therefore, it is reasonable to think that differences between these groups are age-related rather than due to exercise selection and training volume.

### Specific COD Training

With respect to 20 experimental groups performing specific COD training, there were six experimental groups revealing ES > 0.8 (Table 3). These six groups were 16.8-year-old participants with 14 training sessions on average, in comparison to the remaining 14 groups that were, on average, 17.3 years old with 13 training sessions during the intervention period. In other words, the experimental groups revealing marked improvement did not differ much from those revealing less improvement in terms of total training volume and age.

In two studies [51, 91] where the experimental groups displayed very large ES, repeated COD was trained with the same distance and angle used to measure improvement in COD after the intervention. Two experimental groups [95] with ES > 0.8 that trained in small-sided games (which include repeated COD) also conducted repeated COD tests. Taking this into account, training specificity, in addition to age, could explain the occurrence of large ES post-intervention. Younger athletes have less experience with COD training, and training specifically towards the COD test that measures improvement can be advantageous in comparison to groups that are not training specifically enough. This is exemplified by Chaalali et al. [90], who implemented specific COD training with a ball among young soccer players and measured improvement with a 505 agility test. Alongside the number and angle of CODs, the use of a ball complicates the COD task, where the focus on properly handling the ball may prevent the athletes from a rapid completion of the COD task. COD training is effective for young people if the task prevents complications [13].

The lack of specificity can be exemplified by Nakamura et al. [92]. Despite training with 18-m repeated sprints with two CODs, they measured performance with a 70-m COD test with three CODs, resulting in very small to medium ES (ES = 0.13 and 0.5). Both Chaouachi et al. [89] and Young and Rogers [87] used small-sided games in their interventions, showing very small to medium ES post-intervention (ES = 0.67 and 0.2). Differences in percentage increases were also apparent, being 2.5% and 0.8% respectively. The difference between the two studies is that Chaouachi et al. [89] demonstrated medium ES by conducting several CODs with small angles of new direction when measuring COD performance. Since the experimental groups were composed of soccer players in both studies, the results can be explained by the fact that the execution of strength-oriented COD in small-sided games in soccer is not necessary, but more velocity-oriented COD is apparent. Arguably, a different COD test could have been used to track performance post-intervention, since the technique in a COD varies according to the angle and entry speed of the task. Additionally, the same energy systems that are utilized in competition should be targeted during COD training, as exemplified by the effect of repeated COD training upon the ability to reproduce high intensity CODs during competition [21].

### Sprint Training

Six experimental groups performed sprint training, and only two groups revealed ES > 0.8. These two groups trained once a week and were on average 14.6 years old. The remaining four groups with ES < 0.5 were 22 years old on average, and three of the groups trained twice a week. However, the last group trained three times a week for a period of only 2 weeks. de Villarreal et al. [109] point out that small to moderate training frequency (< 2) during a week is most effective to develop physical abilities like sprint, jump and maximal strength. Given that experimental groups that revealed large ES were formed by younger subjects, technical learning effects could explain differences related to performance.

Training groups revealing no ES to small ES performed 20 to 60 m COD tests that included between three and nine CODs of 100 ° or more respectively [72, 77, 93, 98]. During the acceleration phase of sprints, horizontal force is exerted to the ground, followed by vertical adjustment of the body at greater velocity. Bourgeois et al. [13] suggest that runs of fewer than 10-m should be assessed to develop COD ability. Furthermore, the number of changes in direction will influence the specificity regarding sprint training: several COD manoeuvres with exertion of force to the ground in different directions differentiate the muscle work performed in CODs from that in sprints. Previous work points out the need to perform actions in training that are similar to movement patterns performed in competition [21]. Differences in movement patterns are challenging when performing straight-line sprint training to develop COD performance, since the technique is different from COD, even though the acceleration phase is similar [13, 20]. Nevertheless, few experimental groups have assessed sprint training in this context, making it difficult to determine which form of sprint training is optimal to enhance COD performance.

### Combined Training

Of the 24 experimental groups with a combined approach to training for COD performance, only six groups were not composed of soccer players [69, 78, 99, 100]. Gil et al. [102], who showed very large ES in two experimental groups, implemented squat jumps plus COD and sprint training in their training protocol. The result was impressive, considering the athletes were 22 and 23-year-old professional athletes. However, it is worth noting that the athletes were in the pre-season training period. The author states that a significant amount of training, such as small-sided games, was performed in addition to the experimental training protocol. It is also worth noting that previous research in this review has revealed large effects of squat training on COD performance (Table 2). Previous research [35] has revealed that squat jumps with countermovement and an external load of 30–60% of body mass are optimal for exerting maximal power output. This is also a load used in previous studies training for PAP [125], which, theoretically, could enhance performance in CODs and sprints within and after the training session. These are factors that could explain some of the results.

All eight experimental groups revealing large ES had plyometrics in their training protocol. Six of these groups had an average age of 17 or more, whereas the two remaining groups were on average 14.5 and 11.3 years old and trained in COD in addition to plyometric training. The results in relation to the groups displaying large ES can be explained by previous findings in this review, which highlight that plyometric training seems to be beneficial for older athletes due to increased strength after puberty. However, improvements in COD ability could also be a result of little experience, thus making specific COD training a great stimulus for improvements in younger athletes. In contrast, the remaining groups, which display ES > 0.8 and included plyometric training in their training programme were 15.6 years old on average.

The current review explored multiple approaches to training, which included variations concerning age and sex of the athletes, number of training sessions and use of different COD tests to measure performance. Thus, discovering clear trends and conclusions is difficult.

When combining training forms, it is impossible to distinguish between the isolated effects of each individual training form on COD performance. Apart from studies that included plyometric training, several studies revealed impressive results when including sprint and strength training. The benefit of performing combined training is that an athlete can improve multiple determinant factors upon which COD performance depends. In court and field sports, situations occur in which entry speed and angle of the COD task vary, which could support the function of combined training to develop COD performance. With the development of multifactorial determinants, which a COD performance depends upon, the athlete is more primed to fulfil competitive demands in a variety of match situations [21].

### Practical Consequences and Applications

After calculations are made regarding effect sizes, there is no indication of one training form being superior to any other. All training forms contained at least two experimental groups that displayed large ES. Strength and conditioning coaches should aim to optimize training in COD with respect to sport-specific demands and individual players’ role. The effect of different training forms on COD performance can be explained by the force-velocity relationship [126]. Rapid muscle actions detach myosin heads and lead to fewer cross-bridges being activated in sarcomeres. Increased contraction velocity will, therefore, lead to a decrease in muscle force [126]. This review reveals that both strength training and plyometric training are effective training forms in sports that utilize strength-oriented COD, because strength-oriented CODs feature a longer stretch-shortening cycle and greater exertion of muscle force in comparison to velocity-oriented CODs. Sprint training should be implemented to develop velocity-oriented COD in sports where the magnitude and number of CODs are lower, and where sprints followed by COD are performed over longer distances (> 10 m). Velocity-oriented COD is characterized by rapid muscle actions, as occur during sprinting.

Strength and conditioning coaches should be careful when employing CODs over longer distances with several changes in direction. While anaerobic energy systems play a central role in COD performance, practice of several COD manoeuvres over greater distances will target the aerobic energy system more, while making the COD task more complicated [13].

The main consideration when developing COD performance is specificity in the respective sport when organizing the length, magnitude and number of COD manoeuvres in the COD task. Field and court athletes will perform different types of COD during competition; combined training can lead to a wider spectrum of capabilities regarding COD performance. Nevertheless, COD is a situational ability, not a general ability, though increased performance in a single COD task can lead to increased performance in a different COD task [13].

## Conclusion

The ability to perform COD effectively is an important skill in numerous sports, thus making it relevant to investigate which forms of training lead to improvement in this skill. The most important finding in this review is that plyometric training, strength training, sprint training, COD training and a combination of these training forms can be used to develop COD performance. The optimal form of training depends on the types of COD involved in different sports. In general, strength training is sufficient to develop strength-oriented COD, plyometric training is effective in developing both strength- and velocity-oriented COD, and sprint training is beneficial for velocity-oriented COD. Specific COD training can be implemented, but there will be a lack of transferability unless the task is comparable with respect to running patterns in competitive situations. Finally, a combination of the mentioned training forms can be used to develop COD performance if the training is similar to COD in terms of muscle work and duration.

## Data Availability

Please contact author for data requests.

## Abbreviations

CG: Control group
CMJ: Countermovement jump
COD: Change of direction
COM: Centre of mass
DJ: Drop jump
Dom-leg: Dominant leg
EG: Experimental group
ES: Effect size
N-Dom leg: Non-dominant leg
None: Control group not provided
PAP: Post-activation potentiation
Post: Post-test
Pre: Pre-test
R: Repetitions
RDL: Romanian deadlift
RM: Repetition maximum
RSA: Repeated sprint ability test
S: Series
SSC: Stretch-shortening cycle
Tr: Training
